# Development of a Web GIS for small-scale detection and analysis of COVID-19 (SARS-CoV-2) cases based on volunteered geographic information for the city of Cologne, Germany, in July/August 2020

**DOI:** 10.1186/s12942-021-00290-0

**Published:** 2021-08-28

**Authors:** Fabian Schmidt, Arne Dröge-Rothaar, Andreas Rienow

**Affiliations:** Institute of Geography, Ruhr University Bochum, Universitätsstraße 150, 44780 Bochum, Germany

**Keywords:** Geographical Information Systems (GIS), North Rhine-Westphalia, Web GIS, General Data Protection Regulation, Open source, COVID-19

## Abstract

**Background:**

Various applications have been developed worldwide to contain and to combat the coronavirus disease-19 (COVID-19) pandemic. In this context, spatial information is always of great significance. The aim of this study is to describe the development of a Web GIS based on open source products for the collection and analysis of COVID-19 cases and its feasibility in terms of technical implementation and data protection.

**Methods:**

With the help of this Web GIS, data on this issue were collected voluntarily from the Cologne area. Using house perimeters as a data basis, it was possible to check, in conjunction with the Official Topographic Cartographic Information System object type catalog, whether buildings with certain functions, for example residential building with trade and services, have been visited more frequently by infected persons than other types of buildings. In this context, data protection and ethical and legal issues were considered.

**Results:**

The results of this study show that the development of a Web GIS for the generation and evaluation of volunteered geographic information (VGI) with the help of open source software is possible. Furthermore, there are numerous data protection and ethical and legal aspects to consider, which not only affect VGI per se but also affect IT security.

**Conclusions:**

From a data protection perspective, more attention needs to be paid to the intervention and post-processing of data. In addition, official data must always be used as a reference for the actual spatial consideration of the number of infections. However, VGI provides added value at a small-scale level, so that valid information can also be reliably derived in the context of health issues. The creation of guidelines for the consideration of data protection, ethical aspects, and legal requirements in the context of VGI-based applications must also be considered.

*Trial registration* The article does not report the results of a health care intervention for human participants

## Introduction

### Relevance

Knowledge of COVID-19 and its spatial distribution is important because of numerous issues. Cases of infection have been confirmed in almost all countries worldwide [46, 47]. This not only has health consequences for individuals, but also for society, social life, the economy, and the environment, which are directly or indirectly affected by the pandemic. Nicola et al. [39] cited a variety of socioeconomic consequences of the pandemic, affecting all sectors of the economy. In addition to the threat of recession, social distancing and the isolation of individuals through school closures and travel restrictions, for example, have led to lower productivity, which, in turn, is linked to a possible loss of jobs. While the demand for some goods, such as oil, has declined, the market has seen a rising demand for medical products. Panic buying and stockpiling are also increasing demand for food. Governments in some countries have imposed restrictions or are initiating border closures [39]. Furthermore, the public transport frequency is lower in many places [1] or public transport services are used less [3], and air traffic has been reduced to a minimum [34]. Complete industries such as the hotel/hospitality industry have to temporarily stop work [41], and companies can more easily put their employees on short-time work through political decisions to reduce costs [12], while universities and schools have switched to virtual teaching [43] and daycare centers have remained closed altogether. This is accompanied by other additional stresses and problems, such as working in a home office and at the same time having to take care of children [37] or increased case numbers of domestic violence [39]. Major sporting events such as the Tokyo Olympics [57] or the UEFA European Championship have also been postponed by a year [56]. States are developing rescue packages to ease the burden on companies and thus secure jobs in the long term. In the future, this should be done not only at the national level, but also at the European level, such as through joint economic stimulus programs or the introduction of EU coronavirus solidarity funds [23]. Different health systems, treatment options, and interventions by diverse governments [24] are associated with different infection rates and deaths in different countries [46].

### Problem and research needs

The containment and control of COVID-19 is a major challenge [35]. Interrupting and tracing the chain of infection is essential. Various solutions exist for this purpose and can be helpful, but some are questionable or controversial owing to data protection aspects. At the beginning of the pandemic, so-called movement profiles, based on location data, were passed anonymously by mobile phone providers and used to measure the activity of the population and their movement in space, for example, by the telecommunications company A1 Telekom Austria. This approach has its advantages but is criticized as it performs tracking without user consent and follows a methodology that is not very transparent [13]. The Corona data donation app (https://corona-datenspende.de/) is viewed critically because the actual data processing/holding does not correspond to the provisions set out in the privacy policy. For example, it states that data are held or processed exclusively on servers stationed in Germany, and that neither names nor addresses of users can be identified. However, data are transferred directly from the fitness wristband to the Robert Koch Institute’s (RKI) server (without a detour via the user’s smartphone). This provides direct access to the fitness wristband manufacturer’s server, and thus, the theoretical possibility for the RKI to access health data for both the period before the pandemic and the user’s entire name [18]. The Corona-Warn-App commissioned by the German government [14] is based on modern Bluetooth technology (D3PT) [14]. It does not use location data but measures the distances between mobile devices. Users indicate whether they are infected with SARS-CoV-2. If other users are in the vicinity of an infected person, they receive a message on their device [14]. The programming code of the app has been disclosed so that it is an open-source application [14], which increases the transparency of the tool. One weakness is that the data are not directly evaluated at a specific spatial level for the end user. Thus, it is not apparent how infected persons are spatially distributed. Furthermore, the application can only be used with an end device on which the iOS or Android operating system can be installed [19]. Therefore, this article describes a web application that enables the sharing of information about a SARS-CoV-2 infection on a small-scale level, independent of the end device or operating system version, automatically evaluates the collected data, and makes it available to the user. Against this background, data protection and ethical and legal aspects are also considered.

### Research questions

This study aims to answer the following research questions:

- To what extent can a web application be developed based on open-source products for the spatial recording/evaluation of COVID-19 cases in the urban area of Cologne?
- Which data protection, ethical, and legal aspects must be considered when using volunteered geographic information (VGI) in the context of application development?
- It is expected that the development of such web applications is possible. However, its development and data collection are accompanied by various data protection issues. Furthermore, to what extent the tool will be accepted and used by potential test persons or whether valid information can be derived from them and how statements on the distribution of COVID-19 cases within the city area can be made remains unclear.

### Structure of the work

First, this study explains the current state of research as well as the theoretical background of the relevant aspects. This includes basic ideas regarding VGI and suitable areas of application. Furthermore, data protection aspects to be considered in this context are explained, supplemented by associated legal and ethical concerns. This chapter also includes basics on SARS-CoV-2 and COVID-19. Finally, examples of applications that use spatial information in the context of the disease or have been developed in the context of the pandemic are presented. The third chapter, the methodology, deals with the basic architecture of the Web applications developed here and the source data used to collect information. Chapter 4 focuses on the data collected with the help of the developed applications and are evaluated, and the data protection, ethical, and legal aspects associated with them during development and evaluation are considered. Next, the results of the work are critically reviewed, the potential for improvement is highlighted, and limitations are stated. Finally, in the conclusion, the results are summarized in the context of the research questions formulated at the outset.

## Theory and current state of research

### Volunteered geographic information

#### Definition and basics

Goodchild [30] was the first author to introduce the term *volunteered geographic information* (VGI), 2 years after Boulos [11] introduced the phrase-term *Wikification of GIS by the masses* [11]. The first is defined as a process in which numerous private individuals provide and create geographic information. No official institution is involved in the data creation process nor is any formal qualification required. The collection and provision of data are voluntary and vary in accuracy and precision. VGI has a major impact, both on the discipline of Geography, as well as on Geographic Information Systems [17, 33, 42]. VGI is a special case of user-generated content on the web. Prime examples of such generated data include Wikimapia, Flickr, MissPronouncer, and OpenStreetMap [30]. The relevance of geographic information has increased, especially in the case of disasters. The local population may be immediately affected during or after a disaster and may provide data immediately during or after the event, if appropriate tools for data generation and validation are available. Consequently, VGI can also contribute to such applications [31]. Chen et al. [15] pointed out the benefits of VGI under disaster situations. In addition to the high level of detail and the high speed with which data can be made available, the low costs for this process are identified as particular strengths [15]. The authors present the possible mechanisms for quality assurance of VGI. On the one hand, they note that participants in disaster situations should be classified in advance. For example, this could be organizations, the public, experts, or political decision-makers. These roles can, in turn, be linked to specific tasks. The public includes citizens who voluntarily contribute to the data. Experts can verify, correct, and generally assess the quality of the data or determine which data should be used further. Metadata maintenance can also be performed by them. Based on this, organizations must design their approaches and measures and plan personnel deployment. This can involve policy makers, who, in turn, communicate with the public [15]. Furthermore, quality assurance can be promoted using the framework provided by the state. This framework is created, for example, by establishing standards for the collection of VGI data regarding a potential disaster, including data privacy and legal provisions, such as liability. The technical provision of an application or platform for the collection of information is provided by the state, which also provides official geodata as a basis. In the event of a disaster, organizations and the public can use this framework to rapidly collect and analyze data [15]. Engler et al. [22] discussed VGI and web cartography and how the latter represents a significant tool for the management, dissemination, and visualization of these data [22]. Web or cyber mapping was first perceived as an Internet phenomenon in 1997. Since then, maps have developed a value different from that for which they were originally conceived, namely for the localization of places or the representation of a physical environment. Nowadays, they also cover cultural or socioeconomic aspects. Web maps also offer the possibility of linking other media to content (videos and sound) [21]. Professionals can now generate cartographic content [22]. Engler et al. [22] state that cybercartography is a powerful tool for the management, dissemination, and visualization of VGI. This stems from the fact that both components contain common elements. Among these is the multisensory technology. VGI can be linked to audio, images, texts, and videos. Web cartography allows editing and modification of VGI, and new projects can be created relatively easily for an area of interest, thus addressing a wide variety of user groups [22]. Engler et al. [22] also identified legal issues in the context of cybercartography and VGI. Although traditional institutions and their publications are according to state law, numerous matters regarding voluntarily provided geographic information in the context of web cartography need to be legally reconsidered or reevaluated. Furthermore, ethical and data protection issues must also be considered [22].

#### Data protection, ethics, and legal aspects

The collection and exchange of personal data are directly associated with legal, data protection, and ethical issues [7]. These issues are exacerbated by data sharing with third parties. Increasingly, services and devices that can track people’s location or posts on social networks in the form of images, videos, or texts that are directly linked to a location allow sensitive data such as health status or lifestyle habits to be tracked [38, 49]. Mooney et al. [38] highlighted that data protection regulations in the context of information and communication technology can only be pushed through a combination of technological implementations, legal regulations, and social norms [38]. The crucial question is to what extent VGI-based systems can be extended so that the collected data cannot be traced back to individual persons or rather exclusively to their pseudonyms, which they choose themselves when registering. There are also completely unresolved issues, such as how to protect the privacy of individuals who can be identified in photos with geotags. Both individuals contributing data to a VGI project and the users of that information must grapple with ethical considerations. One example is the provision of data by volunteers under crisis situations. Incorrect figures of damages or victims can, for example, result in inappropriate assistance by authorities. Furthermore, the relationship of trust between parties involved can be permanently disturbed, thus calling into question the usability and quality of VGI. From the perspective of the data user, it has to be ensured that voluntarily generated data are only used for the appropriate purposes and are not subsequently sold commercially, for example. This point goes hand-in-hand with data protection law and legal aspects, which means that ethical problems cannot always be clearly separated from those [38]. Sula [54] stated that ethical principles should be considered throughout the research process. This can be done by making all parties aware of their responsibilities, implementing options to edit and revoke data, or providing results in the form of public channels [54]. A crucial role from the legal perspective in the generation and use of VGI is liability [38]. Cho [16] argued that individuals who voluntarily collect data have to be legally protected and should not be held liable as this risk could reduce or destroy the potential and model of VGI [16]. Furthermore, licensing aspects must be considered in the context of VGI [38]. Scassa [48] argued that the interest of collecting VGI differs. Public entities want to motivate citizens to provide VGI to generate data that might otherwise be difficult to collect or verify. Private companies use VGI more as a tool to supplement and correct existing data [48]. When considering legal aspects, these different perspectives of the participants must be considered, namely those of the operator of a VGI application, the person contributing the data, and the users of the corresponding website or application. Notably, data collection and use can also be performed by the same person [48]. When developing their application, operators should take care to include information regarding their license model and other legal aspects for the user. Special attention should be paid to possible third-party providers whose services are used within the application. Because different data are always brought together in a VGI application, different rights of use also have to be observed. If someone implements the Google Maps API to use its base map, the operator is automatically bound by its terms of use [48]. It is also not clearly defined who owns the rights to the generated datasets. Courts may consider the application or a website as a whole to be content worthy of protection, but in turn, not restrict the extraction of data from that site. If an operator wants to prevent data reuse, they must specify this in their own terms of use or take technical measures against it. Participants also have to be wary of who might have access to the data they have entered [48]. It is necessary to analyze which intellectual property rights are attributable to whom. While the author holds the rights to the publication itself, the individual underlying the contributions must, in turn, be differentiated with regard to the rights holders. One way to deal with this is for contributors to cede their rights to the data provided to the author. This approach can be daunting and goes against community principles that open projects must often follow. An alternative is to obtain a non-exclusive license for the data from contributors to distribute the data. This can be time-limited or perpetual. Content that users upload, but to which they themselves have no rights, is problematic. The author is obliged to highlight that such content may not be contributed [22]. For VGI users, it is important to know the terms under which the information may be used. For example, certain Creative Commons licenses prohibit the commercial use of data, while others allow use provided that the source of the data is acknowledged. Finally, Scassa [48] noted that VGI-based applications have a variety of legal issues. Both public and private entities can be the operators of such an application, but they can also be individuals. Operators, contributors, and users should all be aware of their responsibilities, deal with terms of use and licenses, or make them easily accessible and ensure that their content meets their own expectations and perceptions [48]. Data protection aspects are also important when using existing implementations in scripts. The Geolocation API specification includes non-mandatory recommendations that should be considered during implementation, such as providing an interface to easily revoke consent for location tracking [59]. According to a study by Patil and Lai [40], sharing location with others is perceived as information with the highest sensitivity [40]. Doty et al. [20] lists different parameters that can be used to assess the privacy of a user related to the W3C’s Geolocation API (Table 1).

**Table 1:** Parameters for assessing intrusion into the user’s privacy. Own representation according to Doty et al. [20]

Parameter	Description
Aggregation	a. Are the data from users/user groups aggregated? b. Is it possible to draw conclusions about individuals?
Retention periods	a. How long are the data retained? b. Do time limits exist?
Feedback/Transparency	a. To what extent is feedback given to the user? b. How clearly are data protection aspects communicated?
Notices	a. How are collected data made available? b. Which data have to be made available to a user?
User control/consistency	a. Can the user control what data are collected from them?
Minimization	a. Are only the minimum necessary spatial data collected?
Secondary use	a. To what extent are data reused outside the application?
Distribution	a. Can data be sold to third parties?
Purposefulness	a. Is the collection of data justified by the context of the application?

If users of the API violate the provisions of the specification, the integration of those is considered non-compliant but, at the same time, is not associated with negative consequences. Therefore, these normative regulations are not functional requirements [20]. Especially in Europe, the GDPR is important in the context of all data protection issues. The DSK (Working Group on Technology of the Conference of Independent Data Protection Authorities of the German Federal Government and the States 2020) defines data protection requirements in a publication and assigns the corresponding performance targets to them [2]. Table 2 compares these requirements according to regulations and objectives.

**Table 2:** Requirements and performance areas of the General Data Protection Regulation. Own representation according to DSK [2]

Requirements according to GDPR	Performance areas
Transparency for affected parties (Sec. 5 Para. 1 lit. a GDPR)	Transparency
Earmarking (Sec. 5 Para. 1 lit. b GDPR)	Non-linking
Data minimization (Sec. 5 Para. 1 lit. c GDPR)	Data minimization
Correctness (Sec. 5 Para. 1 lit. d GDPR)	Integrity
Storage limit (Sec. 5 Para. 1 lit. e GDPR)	Data minimization
Integrity (Sec. 5 Para. 1 lit. f, Sec. 32 Para. 1 lit. b, GDPR)	Integrity
Confidentiality (Sec. 5 Para. 1 lit. f, Sec. 28 Para. 3 lit. b GDPR)	Confidentiality
Accountability and verifiability (Sec. 5 Para. 2 GDPR)	Transparency
Identification and authentication (Sec. 12 Para. 6 GDPR)	Intervenability
Support in the exercise of data subject rights (Sec. 12 Para. 2 GDPR)	Intervenability
Possibility to correct data (Sec. 5 lit. d GDPR)	Intervenability
Data erasability (Sec. 17 Para. 1 GDPR)	Intervenability
Restrictability of the processing of data (Sec. 18 GDPR)	Intervenability
Data portability (Sec. 20 Para. 1 GDPR)	Intervenability
Possibility of intervention in processes of automated decisions (Sec. 22 Para. 3 GDPR)	Intervenability
Freedom from error and discrimination in the profiling (Sec. 22 Para. 3, 4 i. V. m. ErwGr. 71)	Integrity
Privacy-friendly default settings (Sec. 25 Para. 2 GDPR)	Data minimization, intervenability
Availability (Sec. 32 Para. 1 lit. b GDPR)	Availability
Resilience (Sec. 32 Para. 1 lit. b GDPR)	Availability, integrity, confidentiality
Restorability (Sec. 32 Para. 1 lit. b, lit. c GDPR)	Availability
Evaluability (Sec. 32 Para. 1 lit. d GDPR)	All previously mentioned
Data breach remediation and mitigation. (Sec. 33 Para. 3 lit. d, 34 Para. 2 GDPR)	Integrity, intervenability, confidentiality, availability
Adequate monitoring of processing (Sec. 32, 33, 34 GDPR)	Transparency, integrity
Consent management (Sec. 4 Nr. 11, Sec. 7 Para. 4 GDPR)	Transparency, intervenability
Implementation of regulatory orders (Sec. 58 Para. 2 lit. f und lit. j)	Intervenability

The publication also includes practical measures regarding how the described data protection aspects can be implemented, both technically and organizationally. Notably, these measures are not to be exercised once, but are cyclical processes that are to be tested for their functionality and repeated regularly if successful [2]. Table 3 shows examples of practical measures for fulfilling the requirements or guaranteed objectives set out in the GDPR.

**Table 3:** Exemplary measures for implementing the GDPR. Own representation according to DSK [2]

Requirements of the GDPR	Exemplary measures
Availability	a. Making backup copies of data b. Protection against external influences (malware, sabotage, force majeure, etc.) c. Redundancy of hardware, software, and infrastructure
Integrity	a. Restriction of write and change rights b. Protection against external influences (espionage, hacking) c. Documented assignment of authorizations and roles
Confidentiality	a. Encryption of stored or transferred data as well as processes for managing and protecting cryptographic information (crypto concept) b. Definition of an authorization and role concept according to the necessity principle based on an identity management by the responsible body
Non-Linking	a. Restriction of processing, usage, and transmission rights b. Use of purpose-specific pseudonyms, anonymization services, anonymous credentials, processing of pseudonymous or anonymized data
Transparency	a. Versioning b. Logging of accesses and changes
Intervenability	a. Operation of an interface for structured, machine-readable data for retrieval by data subjects b. Operational possibility to compile, consistent correction, blocking and deletion of all stored data about a person
Data minimization	a. Reduction of recorded attributes of the affected people b. Implementation of data masks that suppress data fields, automatic blocking and deletion routines, and pseudonymization and anonymization procedures

### Case studies for VGI applications for health-related mapping

**Fig. 1:**
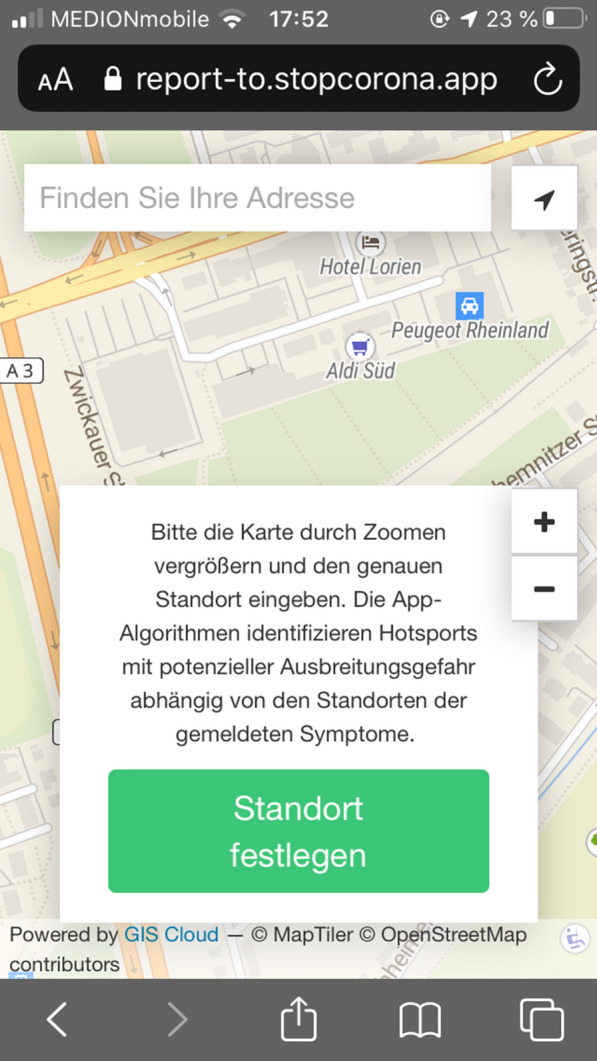
Excerpt of the STOP Corona! App of the GIS Cloud Ltd (German version). (Screenshot from 10.05.2020)

Applications that use VGI data have been developed for various sectors owing to the increasing availability and use of mobile devices and increased network coverage. Examples of VGI usage in the health sector include HealthMap, Sickweather, and the application of the fire department of Ramon Valley, California [10], p. 2. The fire department app of Ramon Valley is designed to optimize the handling of cardiac emergencies [32]. Sickweather is an app that allows users to anonymously share the symptoms of diseases associated with locations. At the same time, social networks such as Facebook and Twitter are scanned for posts in which certain keywords exist, such as bronchitis. If location information is linked to these factors, it is also considered. The data can be viewed by the users on a live map. In addition, the app can send alerts to users when they are in areas where a high number of disease cases have been reported [50]. The website healthmap.org and the app Outbreaks Near Me have been published by researchers, epidemiologists, and software developers in Boston. These apps monitor disease outbreaks and publish real-time data relevant to public health [8]. On the one hand, the app allows users to retrieve reports of disease outbreaks in specific regions, and on the other hand, it acts as an alert system similar to that of the Sickweather application. At the same time, citizens can participate by sharing data on disease outbreaks in their locations [9]. HealthMap has been exclusively implemented with freely available software products, APIs, and libraries [8]. One application that contains pandemics or epidemics developed in the past is EBOLAPP. The app generates and stores user movement profiles using GPS and Bluetooth. In the event of an illness, the user authorizes the doctor to analyze the stored data. Other users receive anonymized warnings that they may have had contact with an infected person based on their movement profiles. German data protection standards apply to the application [27]. The app Aarogya Setu, which is used in India, is designed to warn users if they have come in contact with infected persons. The app works with GPS data. Users also have to enter their name, cell phone number, gender, age, and occupation [58]. In Poland, the Kwarantanna Domowa app was developed. This was used exclusively for home quarantine control. Users are located by the GPS receivers of their terminal devices. The police are responsible for evaluating data. The app users receive text messages at irregular intervals. They have to respond within 20 min and take or send a photo. The app has been mandatory for citizens since April 2020. The generated data will be stored for 6 years. The Austrian app STOP Corona was published by the Red Cross. This involves the exchange of unique user identifiers between smartphones. In the event of an infection, affected persons report directly to the Austrian Red Cross. Subsequently, people who have been in contact with an infected person in the last three days will be notified. The code has been published by the Red Cross and can be viewed at https://github.com/austrianredcross for both iOS and Android and is an open source [58]. A web app was developed by GIS Cloud Ltd. from Croatia. With the app, they aim to collect spatial data on symptoms in the general public on an anonymous and voluntary basis. The goal was to identify hotspots of the virus [28]. In addition to symptoms and location, the time of a post and the user’s IP address are stored in a database. In addition, Google Analytics is used for traffic analysis. The data are subsequently stored on AWS servers in Ohio [29]. Figure 1 shows a screenshot of this application.

**Fig. 2:**
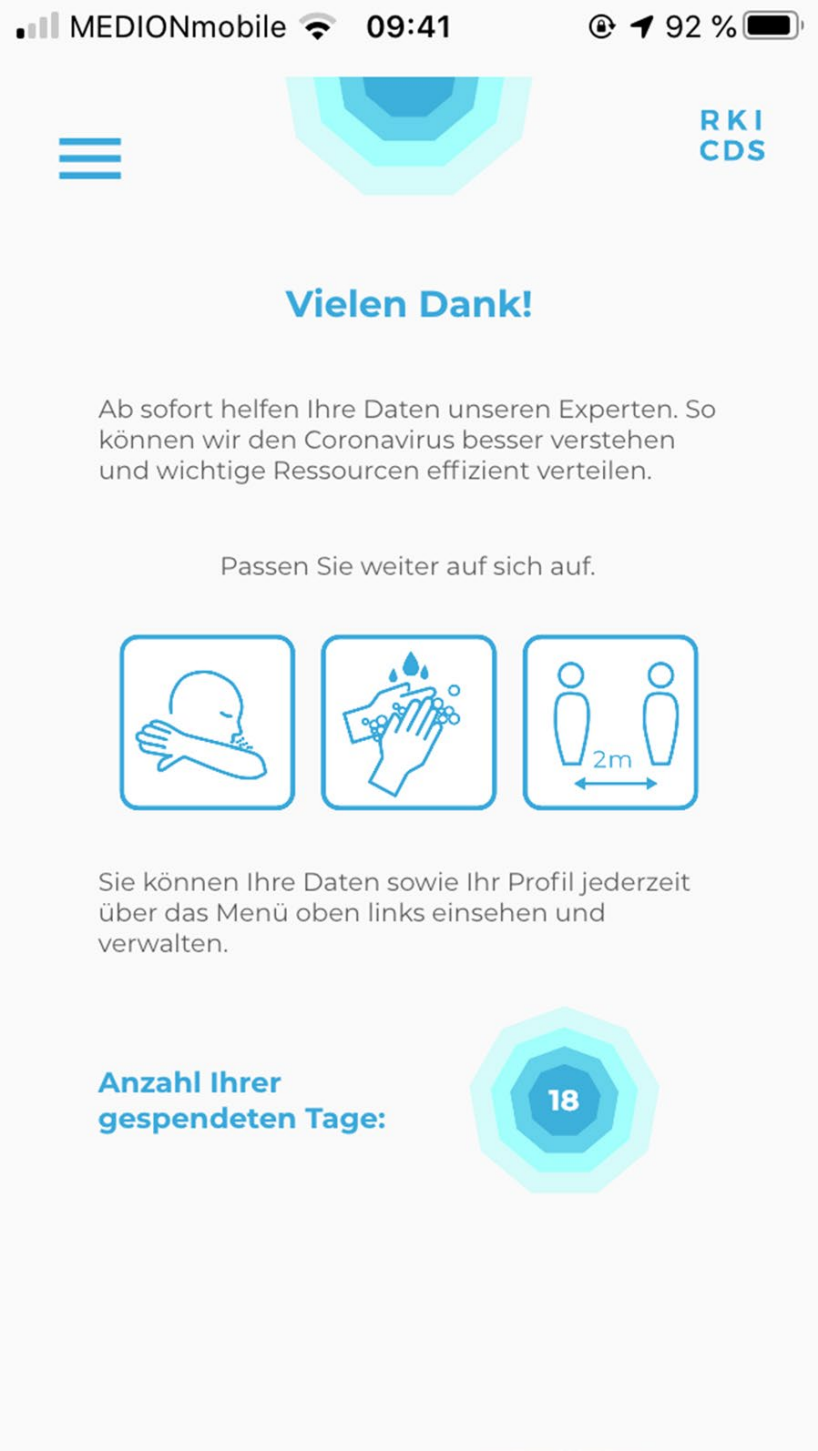
Home screen of the Corona data donation app of the Robert Koch Institute. (Screenshot from 29.04.2020)

**Fig. 3:**
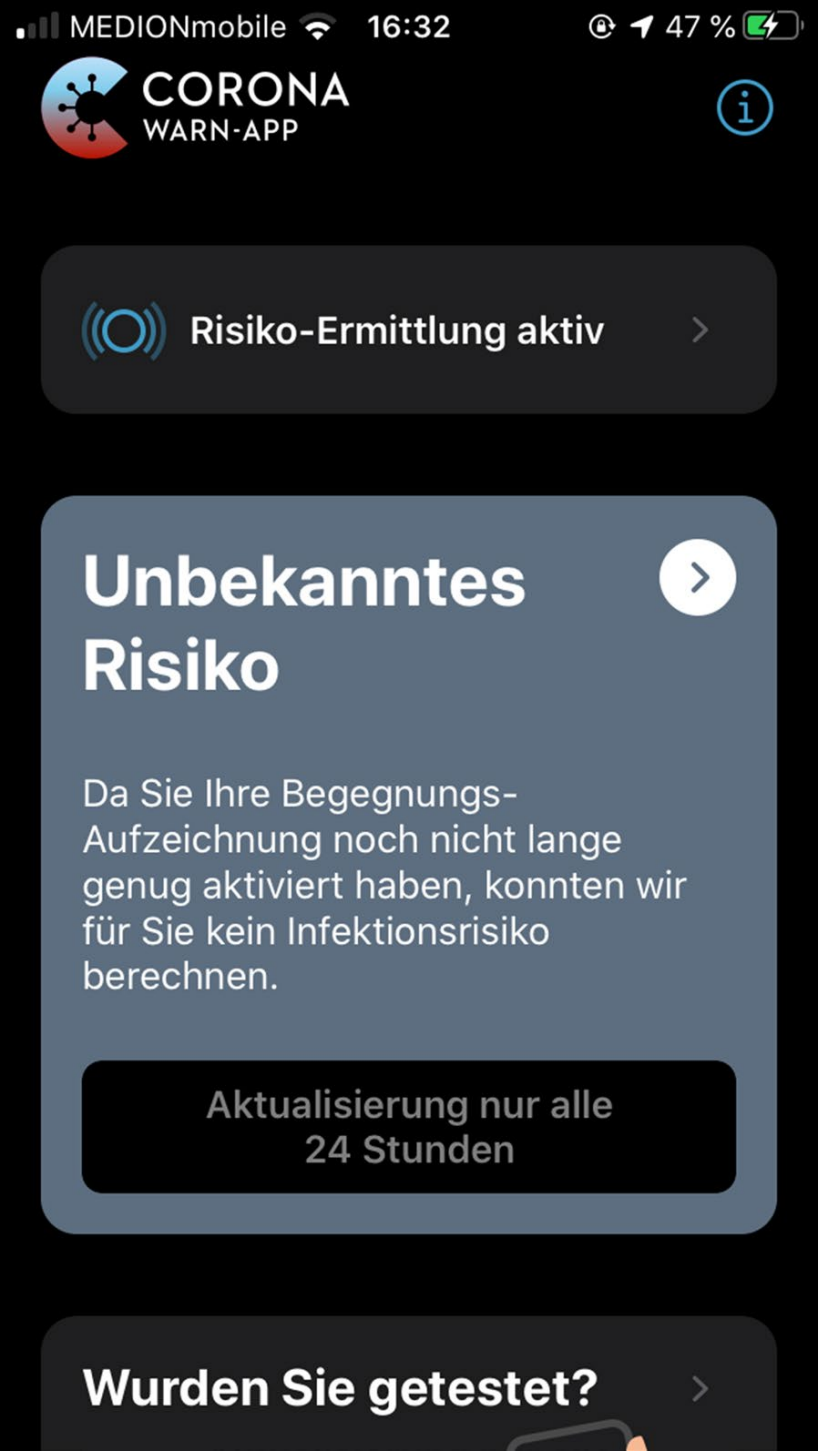
Home screen of the Corona warning app on an iPhone SE. (Screenshot from 13.07.2020)

In Germany, at the beginning of the pandemic, the RKI made a tool for mobile devices available in the form of the Corona data donation app (Fig. 2), with which spatial data can be collected in the context of corona infections. Smartwatches and fitness wristbands can be connected to the app for this purpose. Data were collected on gender, age, weight, height, health and activity, sleep patterns, heart rate, body temperature, and zip code. These were supplemented, for example, using official reporting data. The app monitors parameters and symptoms that may indicate possible SARS-CoV-2 infection. The zip code is used to derive a map showing the number of possible infected persons down to this spatial level. All data are collected anonymously [44]. This is based on a randomly generated ID as a pseudonym, which is automatically initialized during installation and setup and is assigned individually to each user. The app is available for both Android and iOS devices [45]. With the described pseudonym, it is possible to assign data to a user, even in the long term. The user also has the option of viewing, managing, or removing the data provided [44]. However, as described in the Introduction, the application is viewed critically [18]. Other approaches pursue the idea of using non-location-based data for contact tracing based on Contact Tracing Bluetooth Specification by Google. Based on this, the Corona warning app was developed by Telekom and SAP [14]. Figure 3 shows the application on an iPhone SE. The implementation of the application has been limited to end devices with iOS and Android operating systems. Further development, for example, for Windows-based end devices, is not planned [14]. This limitation is addressed in the present study. The developed applications are intended to be platform-independent solutions.

## Methodology

### Basic architecture and source data

**Fig. 4:**
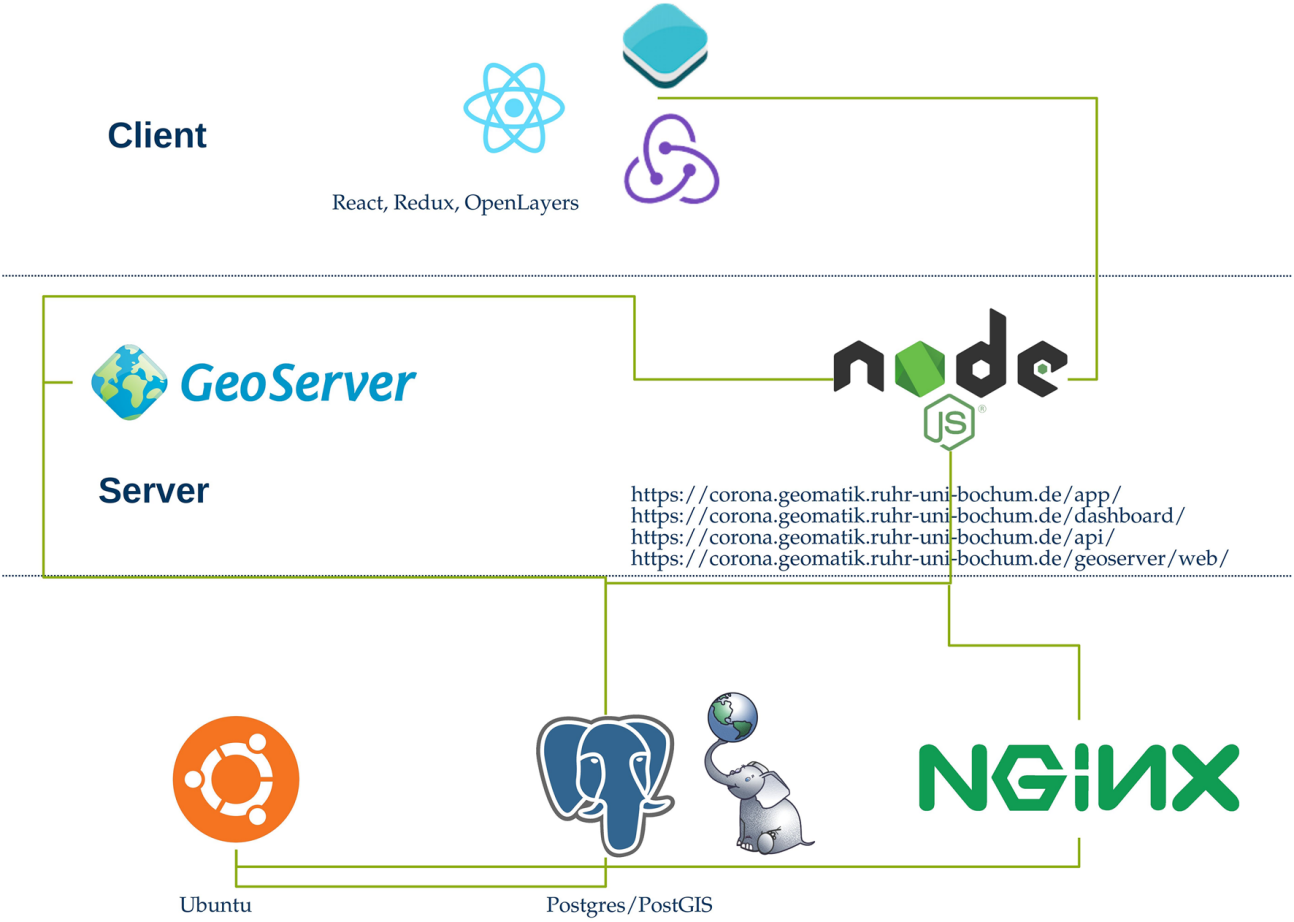
Basic architecture of the applications. (Own representation)

For the implementation of web applications, a certain basic architecture must be implemented. For this, a Ubuntu-driven server was set up. The web server used is Nginx (Engine X), which is used for collection and evaluation. Postgres was used as the database system in this work. Figure 4 shows the basic architecture.

The developed Web GIS serves as the basis for recording COVID-19 cases in the city area. Starting as a basis for data, the house perimeters of all buildings in Cologne are visualized. These have not been digitized but are provided by the *district government of Cologne* (Bezirksregierung Köln) in the form of a shapefile for download. House perimeters are derived from ALKIS objects for buildings [6]. With the help of QGIS, the overall dataset can be tailored to the relevant study area. Additional attributes are added to the existing ones in the shapefile and loaded into the database.

- visited: Boolean indicating whether an infected person visited a house perimeter
- date: date of the last edit
- editedBy: hash value of the user who last edited the respective house perimeter
- editedAt: time when the respective house perimeter was last edited
- sttName: Name of the city district where the respective house perimeter is located

This serves, in particular, for the collection of metadata, which often receives little attention in the context of VGI, and thus, corresponding datasets are often viewed critically. Chen et al. [15] emphasized the necessity of metadata. In total, the trimmed dataset included 322,276 houses. The current dataset is dated 01.01.2020 [6]. The house perimeters are allocated to the respective district via a dataset of the city of Cologne. This is also offered for download as a shapefile and is freely available [53]. Goodchild [30] and Zook et al. [60] identified small local scales as spaces suitable for VGI-based data collection [30, 60], therefore, our study area was limited to the urban area of Cologne. In addition to the city districts, the city of Cologne also allows the download of data on city boroughs [52] and postal code areas [51], which are considered in this work. Taylor et al. [55] identify Cologne as one of the cities that plays a central role in the global economy [55], supported by the spatial and demographic structure of the city, and thus, our findings may be transferable to other major European cities. The RKI also aggregates the collected data in the context of the Corona data donation app at the postal code level [45]. For finer aggregation, this work also uses PLZ8 areas and a land use classification derived from the Urban Atlas. The latter is offered for free download by the EEA and includes polygons derived from satellite data. Land use is assigned to these polygons [25]. The dataset tailored for the city of Cologne includes 9703 polygons. The PLZ8 areas are spatial units defined by the company microm and are derived from official postal code areas. In Germany, there were 82,798 PLZ8 areas. Each unit contained an average of 500 households. Each district is identified by an eight-digit ID, which consists of the actual postal code and a unique three-digit number for that area [36]. For frontend and backend development of the applications for data collection and visualization, knowledge of the following markup- or script languages, frameworks, tools, or concepts is needed:

- Frontend:
  - HTML, CSS
  - JavaScript, Typescript
  - ReactJS, Redux
- Backend:
  - NodeJS
  - Postgres, PostGIS, and SQL
  - Geodata services, Geoserver
  - REST

**Fig. 5:**
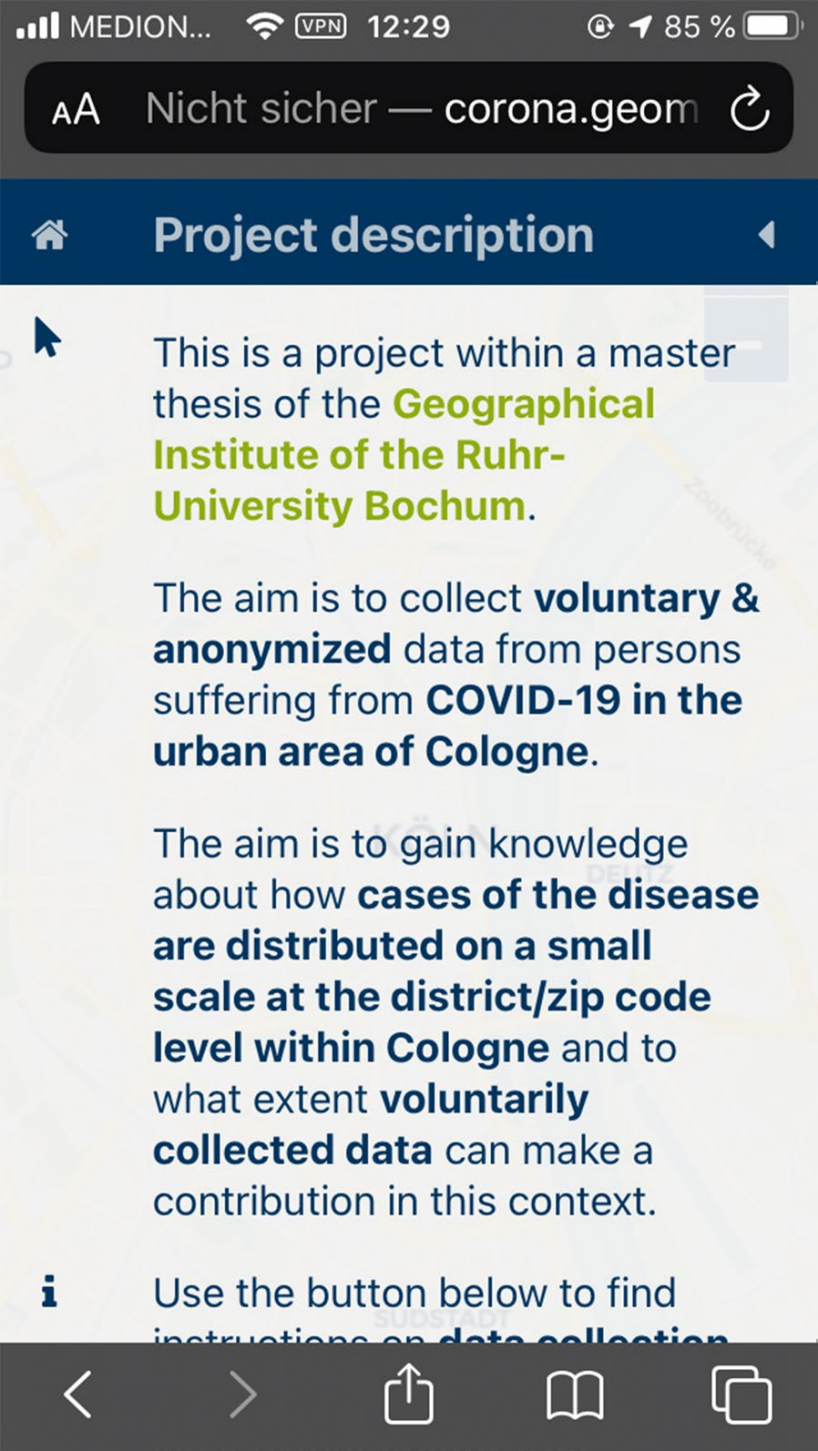
Start screen of the Web application for data collection on an iPhone SE. (Screenshot from 14.06.2020)

**Fig. 6:**
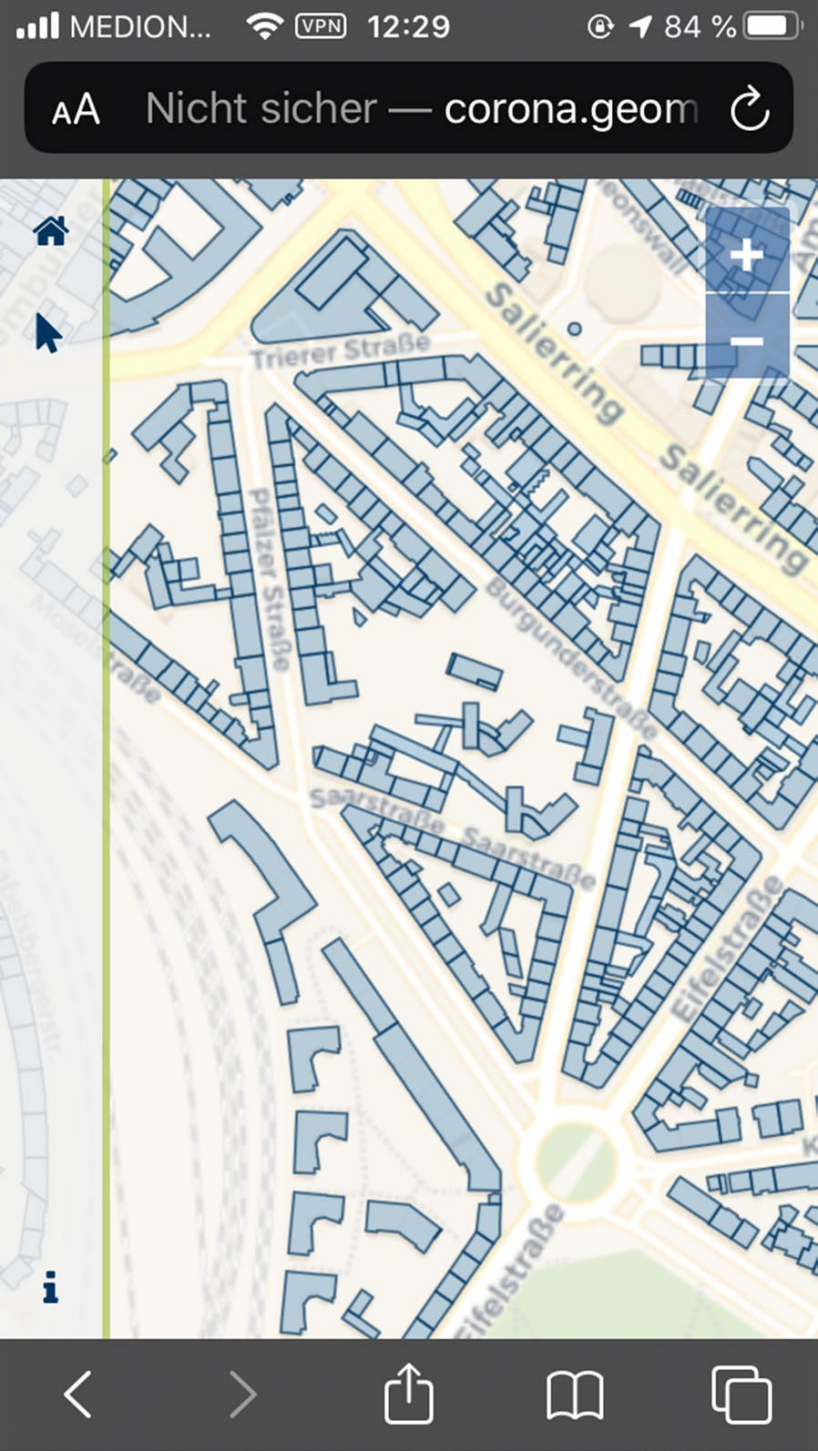
Display of house perimeters in the data collection Web application on an iPhone SE. (Screenshot from 14.06.2020)

Figures 5 and 6 show a screenshot of the developed application for data collection to obtain a visual idea of implementation. Figure 5 shows the start screen with the project description, while Fig. 6 shows how house perimeters are visualized.

### Data collection

The data collection application was implemented using React, Redux, and OpenLayers. It includes a description of the project, privacy notices, the masthead, instructions for data collection, a legend, and the ability to update feature attributes and save these changes in the house perimeter dataset. Participation is voluntary and is intended for individuals who are or have been infected with SARS_CoV-2 and have been in Cologne. They use the application to determine the buildings they visited in the period before diagnosis. The visited attribute is updated by building selection. In the menu, the value of the selected house perimeter can be changed via a button (false for unvisited, true for visited) and saved. By default, all house perimeters have false default values. The application identifies a user via a 128-character token that is generated on the server side when the page is called up for the first time. This token is stored in the browser’s local storage and reused when the app is called up again later. However, the user can delete this in his browser settings at any time. Furthermore, the application offers the user the option of renewing or deleting the token with a click. Thus, any change can be made under different pseudonyms or completely anonymously. The token is also bound to a specific browser. For example, if the user calls up the application with Safari, then with Chrome, and makes changes to the data set in each case, separate tokens are generated for each browser, and it is not possible to assign them to one another. This gives the user control over the extent to which the data are collected from them. This addresses demands from the W3C [59] to implement user interfaces that allow easy revocation of consent, although the committee’s original demand was related exclusively to the geolocation API.

User control is an aspect that Doty et al. [20] also cite to assess the invasion of a user’s privacy. According to the GDPR, the use of pseudonyms or anonymization is an exemplary measure to ensure non-linking. Simultaneously, there are time limits on the retention of data (for the period of this work), and it is clearly communicated that the data may not be further used by third parties. The use of the Geolocation API is waived because the exact position of the user during data collection is irrelevant, and this corresponds to the demands of Doty et al. [20] that only the necessary spatial unit for a purpose should be transmitted [20]. This also follows the requirements for the GDPR, which calls for data minimization [2].

### Data evaluation

**Fig. 7:**
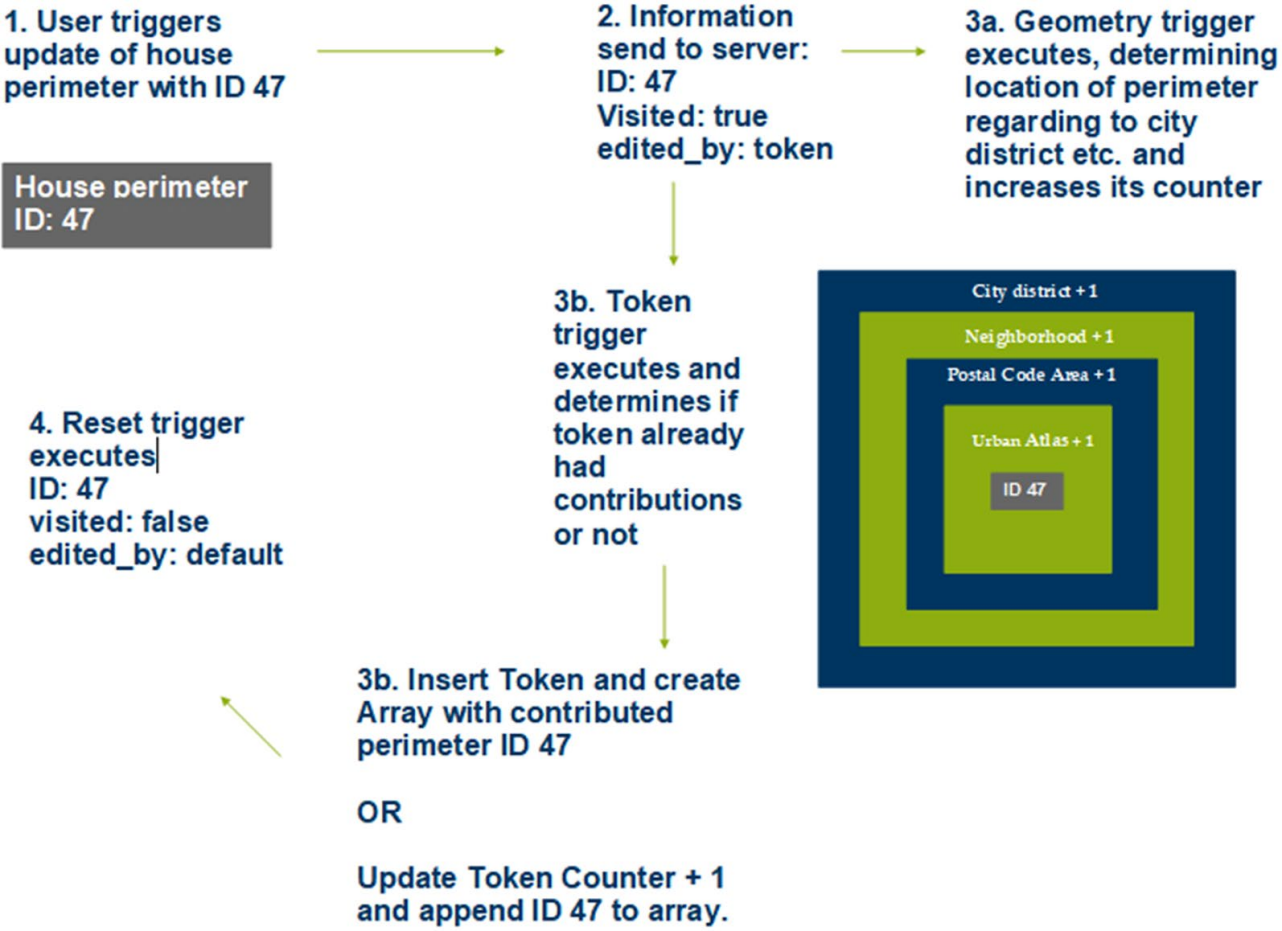
Process of evaluating a house perimeter after updating the information of a user. (Own representation)

Automated evaluation is performed exclusively in the Postgres database. After a user updates the visited attribute of a feature and saves a house perimeter, the house perimeter table triggers various functions. These first check the location of the saved record. This involves determining in which city district, city neighborhood (Listing 1), and postal code/PLZ8 area the affected house perimeter is located. It also checks the area in which the dataset is located within the land cover classification according to the Urban Atlas 2018. Furthermore, the token of the user, if available, is stored in a table, as well as the ID of the house perimeter that the user has updated (Fig. 7). From the perspective of data protection and ethics, it is important that the evaluation is carried out separately from the input data record, so that it is not possible to identify which house perimeters the visited attributes have already been updated in the data collection application, thus preventing third parties from drawing conclusions about individual users. As an additional security measure, 128-digit tokens are stored in the database in an MD5-encrypted form. Thus, contributions can still be assigned to a user, but the token generated for the client in the browser is never available in plain text and can only be reconstructed with the expenditure of enormous computing power. It should be mentioned that the token may have been changed by the user and thus contributions can no longer be assigned to a specific user. The ID of the edited house perimeter is also extended by random numerical codes. Two digits are inserted before and after the actual ID, such that the house perimeter with the original ID 56 is stored in the corresponding Postgres table with 285647, for example. This measure is implemented with regard to the requirement for confidentiality (encryption of stored and transferred data) according to the GDPR and also ensures integrity (protection against external influences). Listings 2 and 3 show the function for generating a random ID and the query that enables assignment to the original ID, respectively.

**Listing 1:**
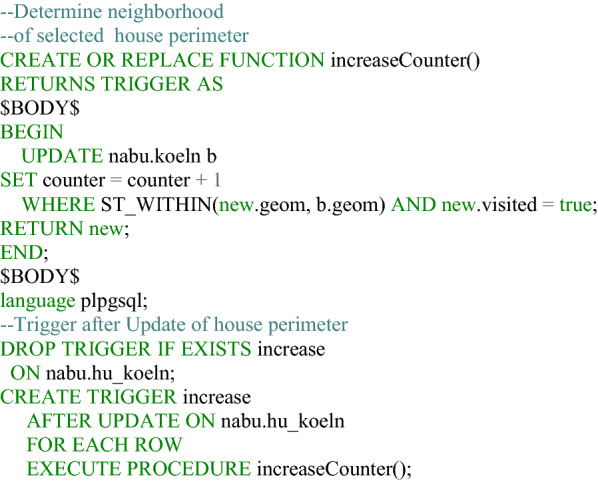
Function to determine the location of a house perimeter in an urban area

**Listing 2:**
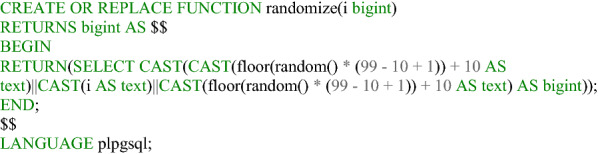
Function to generate random ID in Postgres

**Listing 3:**
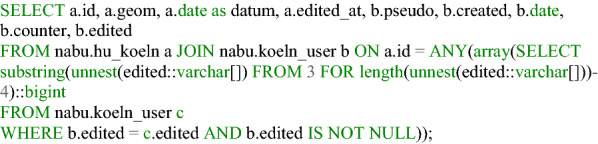
Decryption of the random ID and assignment of the original ID to identify the edited house perimeters of a user

Backups of the database are created daily to secure the application. The data statuses of the last three days are maintained. Older data are deleted automatically. In addition, the server receives a daily report of the current database as a csv file, broken down by the different spatial units. Furthermore, as an additional security instance, an export of the database tables as a shapefile takes place every day to be able to immediately process geodata locally, for example, with QGIS. These practices are intended to meet or support the requirements of GDPR for availability.

**Fig. 8:**
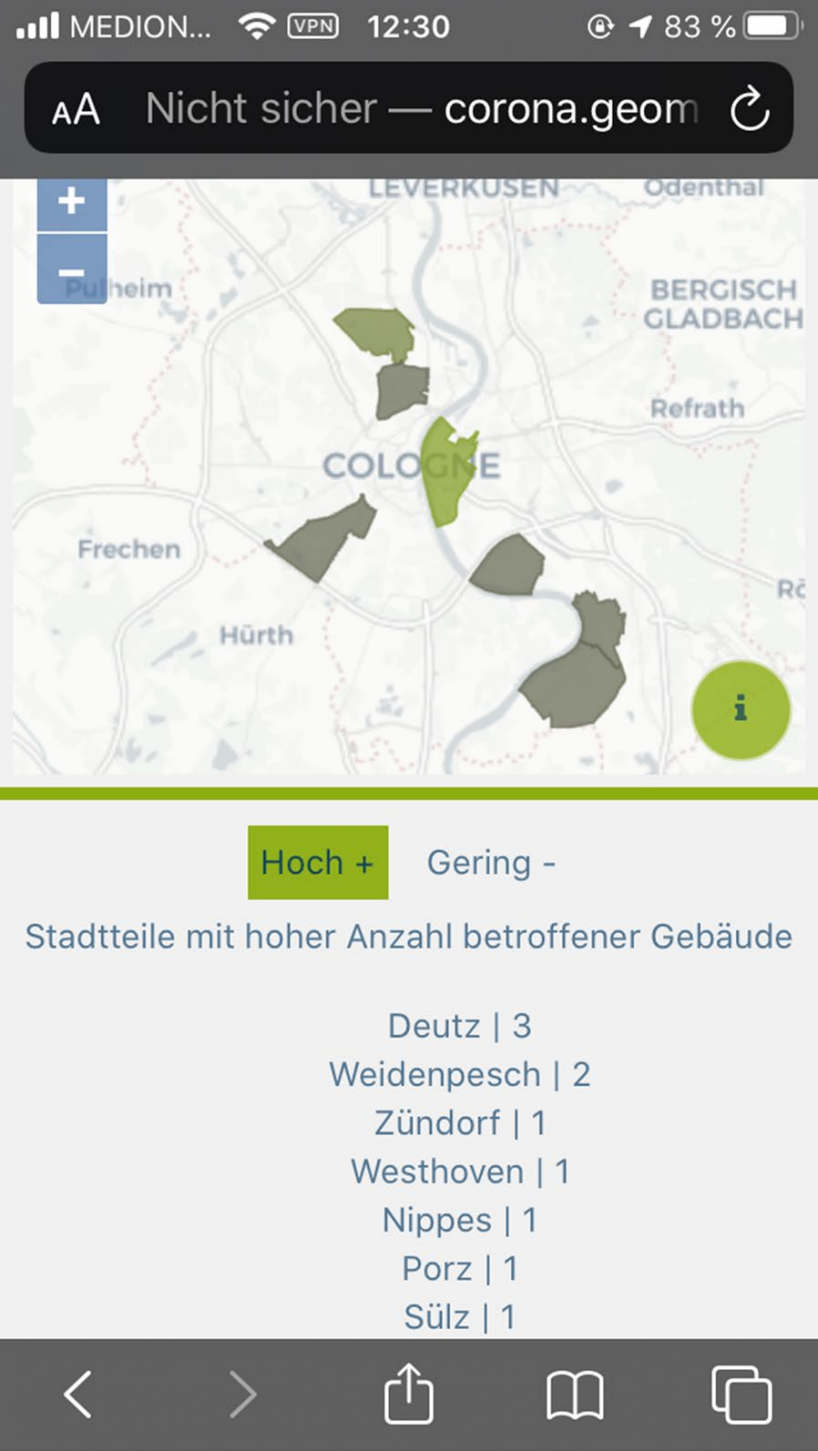
Web application for data evaluation on an iPhone SE I. (Screenshot from 14.06.2020)

**Fig. 9:**
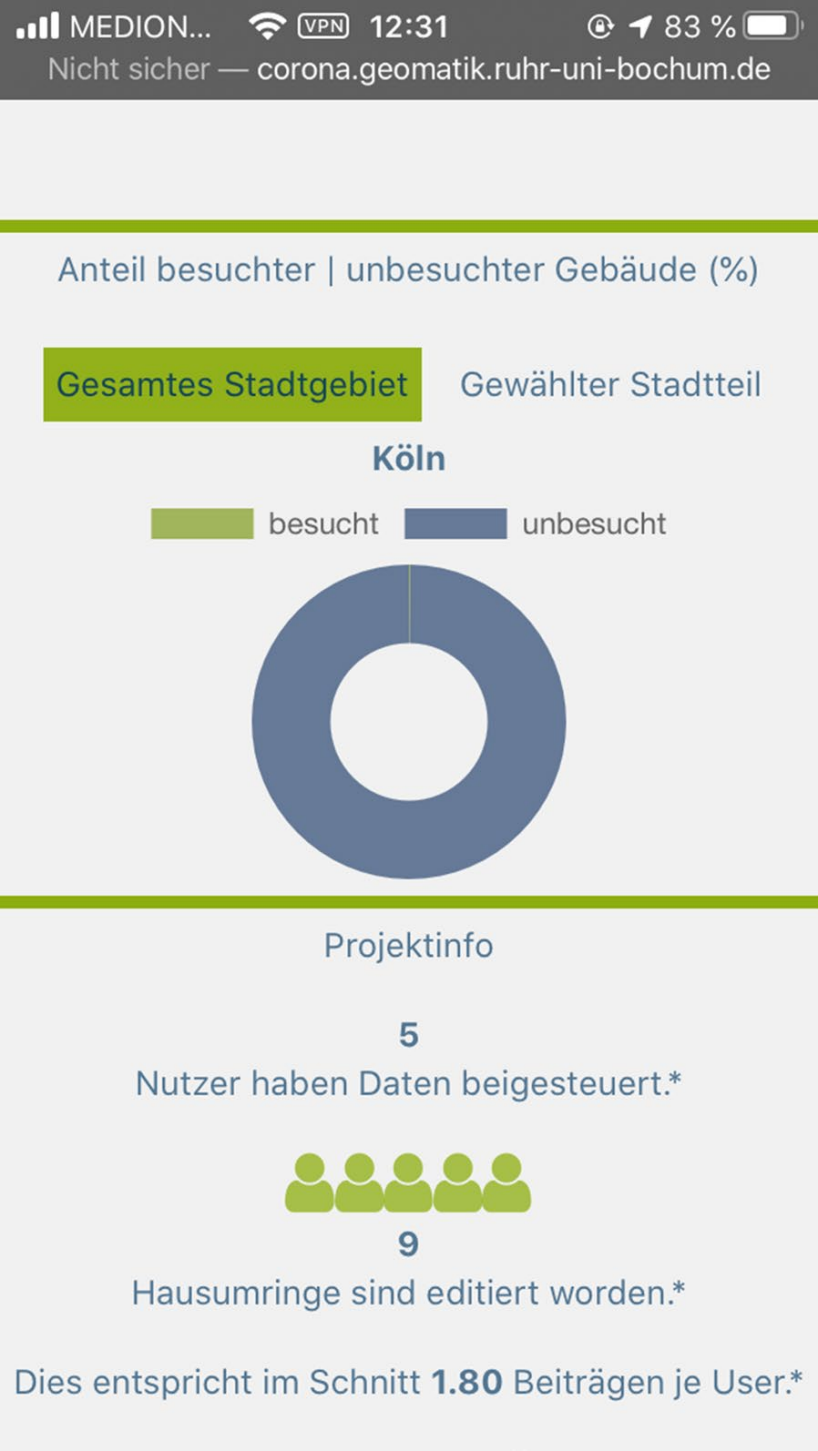
Web application for data evaluation on an iPhone SE II. (Screenshot from 14.06.2020)

The collected data were visualized using a React application and a REST interface. Figures 8 and 9 show an example of a section of the application for data evaluation. Based on NodeJS, the REST interface queries data from the Postgres database using SQL commands. Each implemented endpoint contains a different SQL query and returns different data formats, for example, GeoJSON, JS arrays, or JS objects. The return values of the endpoints are visualized in the form of tables, maps, and charts using React and OpenLayers. Additionally, general information regarding the project participation and its progress has been published, and a Twitter feed is included. The user can also manipulate certain endpoints and query-specific data for a district. Listing 4 shows an excerpt of the code for such an endpoint. The direct publication of collected data thus fulfills the aspect of transparency cited by Doty et al. [20] and the ability to provide immediate feedback to the contributor and provide collected data [20]. This also corresponds to the demands of Sula [54] that results should be made available in the form of public channels [54].

**Listing 4:**
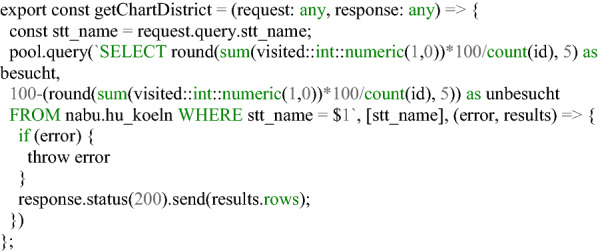
Code snippet for the SQL query in Node via a REST interface

To assess the quality of the collected data, buildings were classified according to the official building/structure functions of the ATKIS (the Official Topographic Cartographic Information System) object-type catalog Basis DLM of the Working Committee of the Surveying Authorities of the Laender of the Federal Republic of Germany [4]. All the applications were developed transparently. For tracking purposes, the code of the web apps, as well as the API, is made available on Github. This versioning and logging follows exemplary measures for implementing the GDPR described in Chapter 2 [2]. Publishing via Github has also been chosen by the Austrian Red Cross as a transparency tool during development [58]. Table 4 lists the associated repositories for these applications. The repositories do not include a Docker container with the corresponding Geoserver or Postgres setup.

**Table 4:** Applications and deposited Github repositories. Own representation

Application	Github-repository
Data collection	https://github.com/fschmidt56/ma_app
Data evaluation	https://github.com/fschmidt56/ma_dashboard_client
API	https://github.com/fschmidt56/ma_dashboard_server

## Results

### Data collected

A total of 20 pseudonyms contributed data to this project during the testing period between July 3, 2020, and September 3, 2020, using the implemented Web GIS. In this process, 50 of the 322,276 house perimeters in the base dataset were updated. All participants contributed data under a pseudonym during the survey; thus, no contributions were made anonymously. Twelve house perimeters, however, were updated twice or more by users; thus, 38 different records were effectively updated. Users contributed data on 14 different days, 10 days in July and four days in August. Table 5 shows the MD5-encoded pseudonyms and their number of contributions, as well as the adjusted number of contributions after eliminating duplicate or multiple selections. In the remaining sections of the chapter, only the adjusted numbers (38 houses per meter) are considered. Tables 6, 7, 8, 9, 10 show the evaluation of the contributions at the different spatial levels as well as the classification of the house perimeters by the ATKIS object-type catalog. The latter may be of interest for further studies, for example, to investigate in which type of buildings infected persons stayed more often or less often.

**Table 5:** MD5-hashed pseudonyms and the associated number of posts. Own representation

Pseudonym	Number	Actual number
09aa4ae9c8bd60bc8b95cef51ee3bf1a	1	1
387e007d70301b0a8d85622ba8b31f04	3	1
7cbbe7d85c04f0e71dd0edefb3f5c318	3	2
855863e96df9380fe5abc40d6c348f51	6	5
e6e8f30224da20ef995dcaef89be8d31	7	4
df51654155725112ea2ffd7ada181e8c	3	1
5bd430ed6850645bc481af7f1a199db8	4	3
106188a01eb1a1aea046cfeec49f29b3	5	3
6444030e41771e099aea2a1bd18d4caf	1	1
672e6912b00b57149199b98a88ee7579	1	1
580841499d0f600773e32ca4ef22062e	3	3
1a24844549971f32c7cc52a5901c3d6d	2	2
4785aa1657b4b3f8f74742430fe64d34	2	2
caf31a6d04b663e11ab1cf1627e294f6	2	2
299a97073114c4b9e7965dd816f48b4e	2	2
67bc57eea1b641b2b33e2ff00efd0790	1	1
f2466edc1b8c780c492eca772a93d088	1	1
6b552b43e324962701aed360740ddcee	1	1
af4dc7b6e5ede0b11894fd4b90eefb42	1	1
ccc917eae5a5b3d73bec22cd04840563	1	1
Discrepancy rate	0.76

**Table 6:** Updated house perimeters by city districts. Own representation

District	Edited house perimeters
Chorweiler	0
Ehrenfeld	1
Innenstadt	19
Kalk	1
Lindenthal	12
Mülheim	0
Nippes	3
Porz	1
Rodenkirchen	1

**Table 7:** Updated house perimeters by neighborhoods. Own representation

Neighborhood	Edited house perimeters
Altstadt Nord	4
Altstadt Süd	6
Braunsfeld	1
Deutz	4
Ehrenfeld	1
Lindenthal	6
Neustadt Nord	2
Neustadt Süd	3
Niehl	3
Müngersdorf	1
Rodenkirchen	1
Sülz	4
Wahnheide	2

**Table 8:** Updated house perimeters by postal code. Own representation

Postal code	Edited house perimeters
50931	5
50676	5
50667	4
50679	4
50937	3
50735	3
50674	3
50678	2
50933	2
50939	1
50677	1
50935	1
50996	1
50823	1
51105	1
51147	1

**Table 9:** Updated house perimeters by land use classification units from the Urban Atlas. Own representation

Land use classification unit	Edited house perimeters
Continuous urban fabric (S.L.: > 80%)	27
Discontinuous dense urban fabric (S.L.: 50%—80%)	3
Industrial, commercial, public, military and private units	8

**Table 10:** Classification of selected house perimeters based on the ATKIS object type catalog of AdV [4]

ID	Neighborhood	ATKIS identifier	ATKIS termination
980	Deutz	31001_1010	Residential house
8384	Sülz	31001_1120	Residential buildings with trade and services
35525	Niehl	31001_1120	Residential buildings with trade and services
44750	Altstadt/Süd	31001_3021	General education school
46562	Niehl	31001_1010	Residential house
49123	Sülz	31001_1120	Residential buildings with trade and services
94262	Neustadt/Süd	31001_1120	Residential buildings with trade and services
98265	Deutz	31001_2071	Hotel, motel, pension
98375	Ehrenfeld	31001_1010	Residential house
103153	Lindenthal	31001_1010	Residential house
103943	Kalk	31001_3010	Administration building
103992	Wahnheide	31001_1010	Residential house
104290	Neustadt/Nord	31001_1120	Residential buildings with trade and services
104476	Lindenthal	31001_1120	Residential buildings with trade and services
108831	Niehl	31001_2000	Building for economy or commerce
110188	Altstadt/Nord	31001_2070	Building for accommodation
371809	Braunsfeld	31001_1010	Residential house
372301	Deutz	31001_3041	Church
375406	Altstadt/Nord	31001_2010	Buildings for trade and services
377393	Altstadt/Süd	31001_1120	Residential buildings with trade and services
413621	Neustadt/Süd	31001_1010	Residential house
423773	Neustadt/Süd	31001_1120	Residential buildings with trade and services
496656	Sülz	31001_1120	Residential buildings with trade and services
502988	Lindenthal	31001_1120	Residential buildings with trade and services
529368	Altstadt/Süd	31001_1120	Residential buildings with trade and services
531633	Altstadt/Nord	31001_1120	Residential buildings with trade and services
542038	Rodenkirchen	31001_1022	Retirement home
550509	Altstadt/Süd	31001_1010	Residential house
550520	Altstadt/Nord	31001_2020	Office building
551728	Altstadt/Süd	31001_1010	Residential house
553959	Sülz	31001_1120	Residential buildings with trade and services
581753	Neustadt/Nord	31001_1120	Residential buildings with trade and services
586091	Deutz	31001_2055	Kiosk
596945	Lindenthal	31001_1010	Residential house
603653	Lindenthal	31001_1010	Residential house
604681	Altstadt/Süd	31001_2020	Office building
611929	Müngersdorf	31001_3060	Buildings for social purposes
1081087	Lindenthal	31001_1010	Residential house

### Data protection, ethical, and legal aspects

Various data protection, ethical, and legal aspects have been considered in the development and implementation of the applications and the evaluation of data collected. In particular, the measures and recommendations of the DSK [2] were considered. Each application includes an imprint that contains contact persons or persons responsible for the content of the page. To ensure availability, scripts for making backup copies of the database, for example, have been implemented. In addition, IPs whose server requests have been suspicious are blocked with the help of a blacklist. To maintain integrity and minimize data, roles and users are defined for the database and geoservers, with only the necessary write and change permissions assigned. The confidentiality required by the DSK [2]was ensured by encrypting the updated data records. For non-concatenation, pseudonymization and anonymization procedures were implemented with the help of tokens, which also met the requirement for data minimization. Transparency was ensured by open versioning of the source code on Github (see Table 4). Intervenability was not considered in the applications. From a legal viewpoint, the use of the collected data is prohibited for third parties, and an evaluation of all information is only allowed in the context of this work. This goes hand-in-hand with the ethical requirements that data may not be sold commercially afterwards. Furthermore, with regard to ethical aspects in this work, special emphasis has been placed on the fact that updates cannot be traced back to specific individuals.

## Discussion

**Fig. 10:**
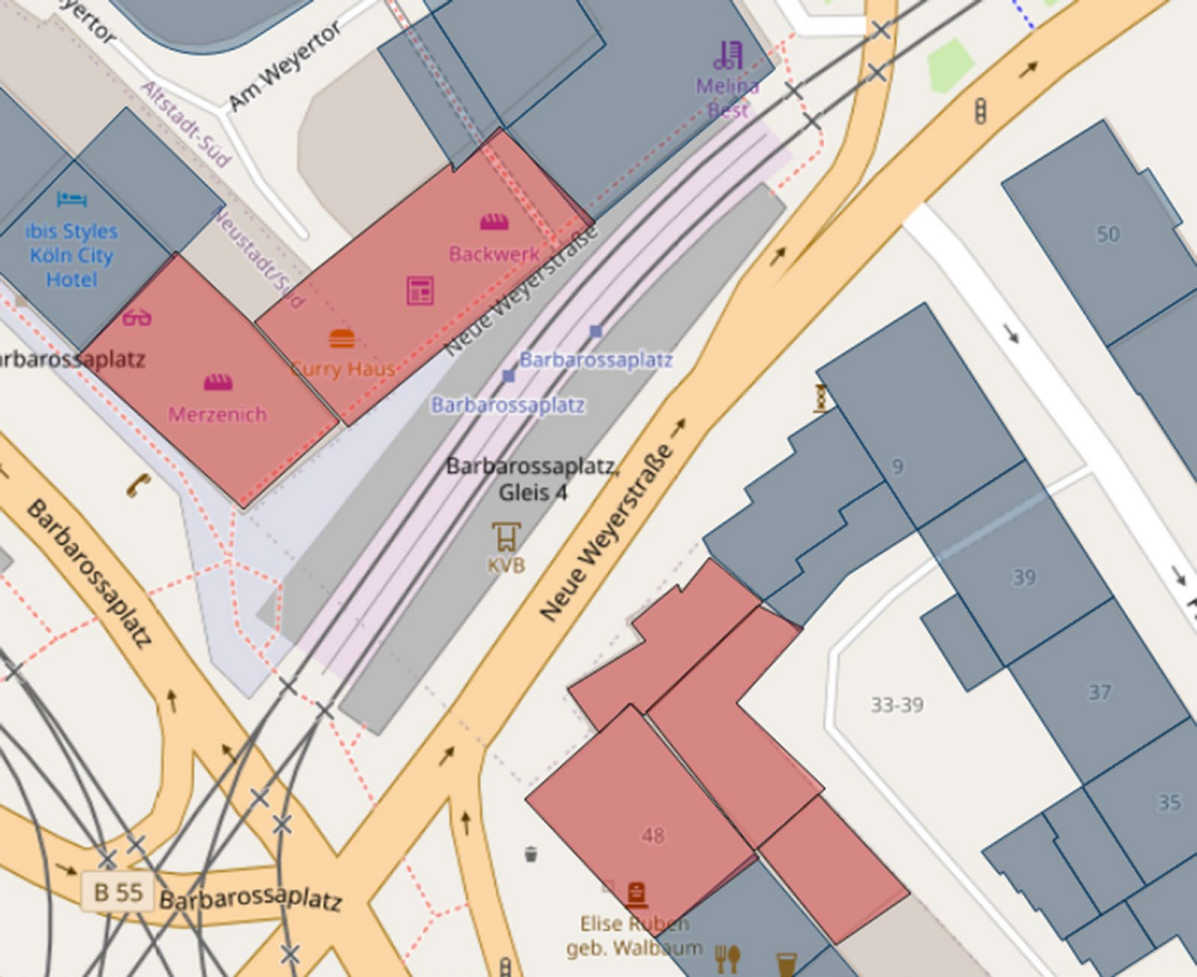
Data gap in the initial data set. Blue: initial data, red: missing data. (Screenshot from 14.09.2020)

**Fig. 11:**
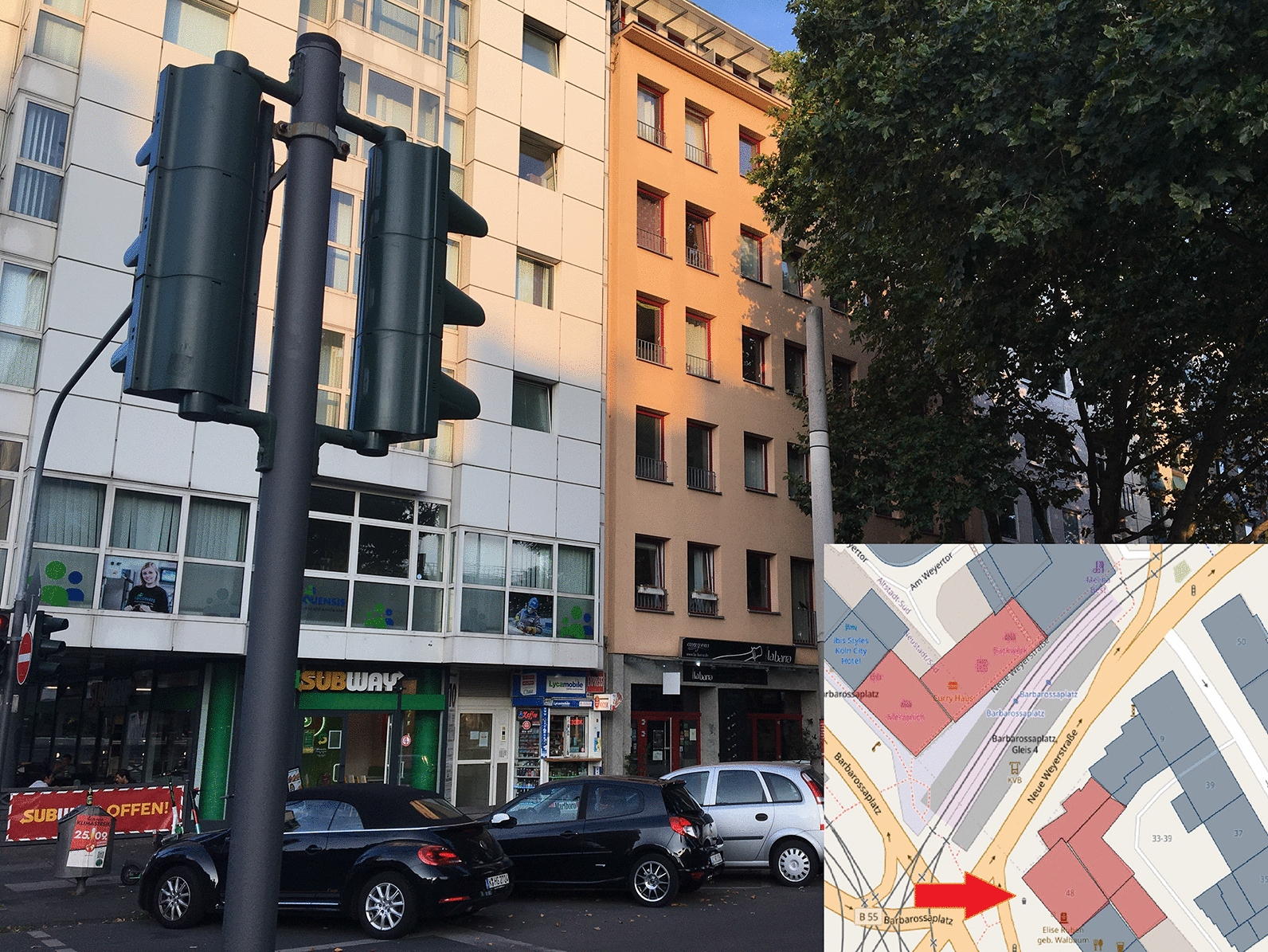
Data gap in the initial data set II. (Own photo from 20.09.2020)

**Fig. 12:**
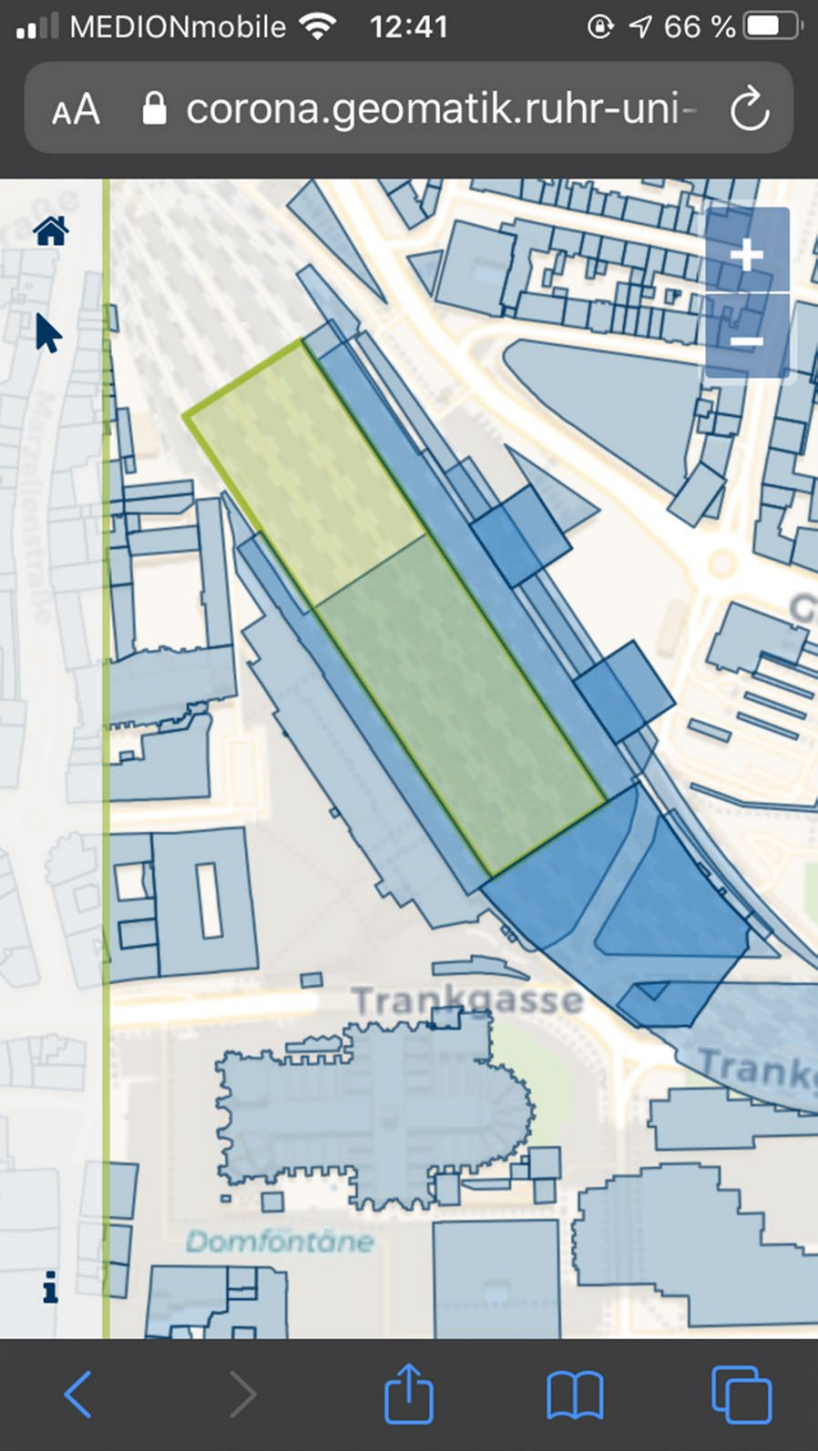
Selected house perimeter of the Cologne Hbf on an iPhone SE. (Screenshot from 05.07.2020)

**Fig. 13:**
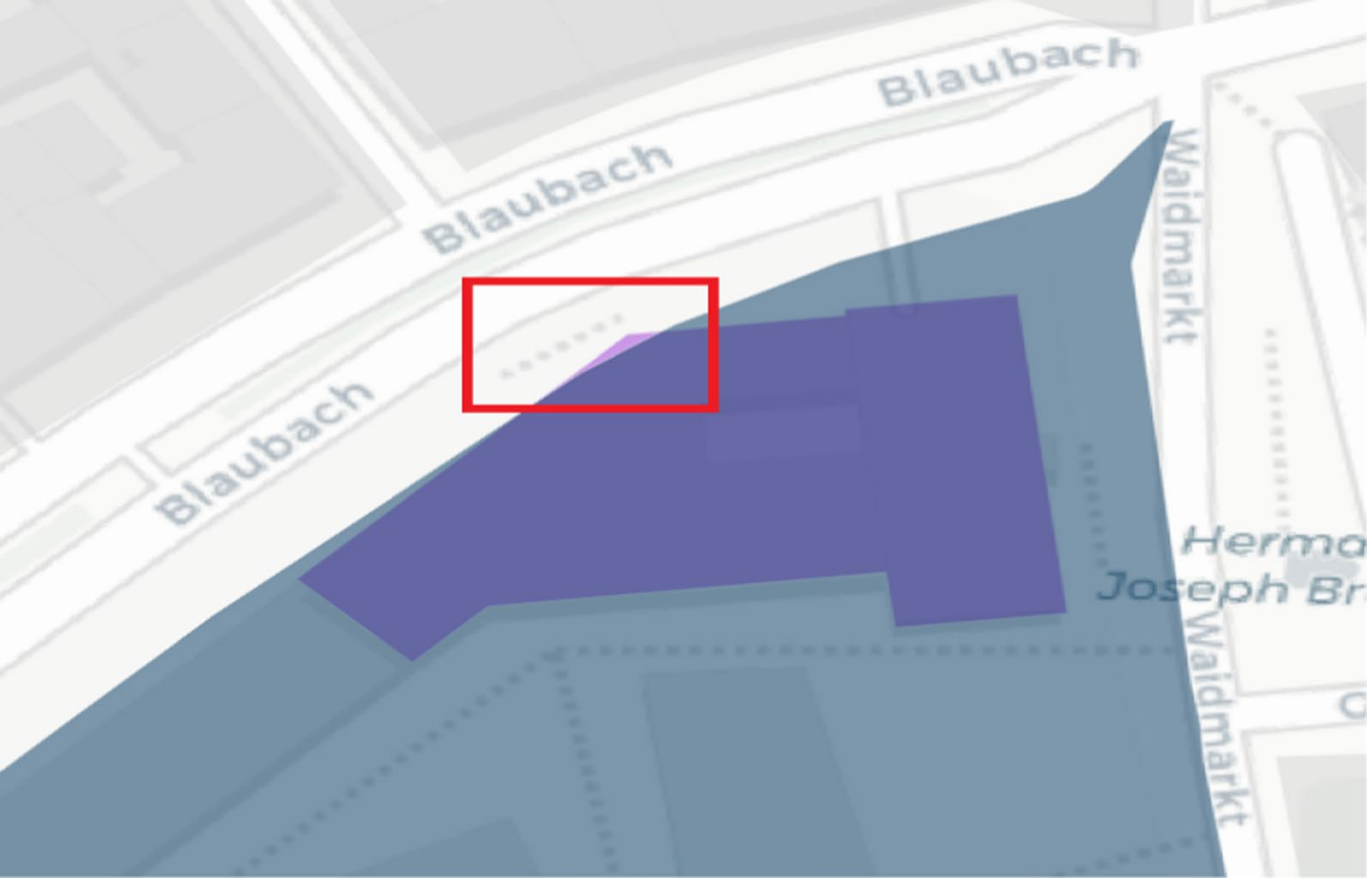
Difficulty in determining the position of a house perimeter due to overlap with a polygon of the Urban Atlas. (Screenshot from 07.09.2020)

The development of applications for data acquisition/analysis with the help of open-source products and open data has been possible without any challenges using the aforementioned frameworks, languages, and components. When implementing these applications, a large amount of data protection and ethical considerations must be taken into account. IT security plays a significant role in this process. From the viewpoint of data protection, VGI generally already makes a contribution through its voluntary nature because users decide on their own responsibility whether and which data they want to contribute. Infected persons can specify all the buildings they visited, but they do not have to. For example, one’s home or workplace may be places that the participants do not want to specify. This may be a variation of the Not in my Backyard phenomenon, as described by Engler et al. [22]. Simultaneously, the data collection application offers the possibility of anonymously or optionally contributing data via pseudonyms. However, this data protection aspect is offset by the loss of information. If data records are collected anonymously or via a pseudonym, the total number of individuals who contributed data can no longer be traced. It is, therefore, not possible to state how many people have updated data records. Furthermore, it is no longer possible to determine the validity of certain data. If a single, identical building is repeatedly selected anonymously, these incorrect contributions distort the overall result. Here, an additional control instance has to be considered, for example, for each browser session, each house ring can be selected only once. Alternatively, captchas can provide a remedy, but they reduce user experience and may not be suitable in disaster situations. Many requirements of the GDPR were considered during development (data minimization, integrity, non-concatenation, transparency, availability, and confidentiality). The requirement formulated therein for intervenability, for example, a possibility to permanently delete all contributions or to correct information, has not been implemented in the context of the work, but should definitely be considered in a new or further development of the application. For this purpose, new functions must be added to the code. However, with the help of OpenLayers, it is possible to provide appropriate workflows and implement transactions for updating and deleting records. This can be done based on WFS services that retrieve data from the Postgres database tables. This basis is already provided by the current development of applications for data collection. However, the anonymization or pseudonymization procedure implemented in this study can be problematic in this context. Anonymous contributions cannot be assigned to any user, so subsequent updates of the data are impossible. The same applies when the pseudonym is changed. The development of a registration or login system can provide a remedy. However, this involves increased programming efforts and further data protection issues, such as secure and encrypted storage of access data. Simultaneously, registration processes can reduce the willingness to contribute data as this creates additional work, and login data are stored permanently. It also contradicts the terms of confidentiality, data minimization, and non-concatenation. In general, there are no explicit guidelines for the implementation of data protection and ethical or legal measures. Therefore, it may be an option to develop appropriate guidelines for the implementation of corresponding measures in the development of VGI applications. The publication of DSK [2] used in this work, which contains exemplary measures, can serve as a guide here. However, these are not specified for VGI platforms. Another point is that many house perimeters of infected persons may not be recorded because they are regarded as private spatial units by the persons concerned. It could be helpful to provide such an application directly to an institution. In addition, access should be enabled only for those affected or infected in the event of illness. The inclusion of a state institution could increase acceptance and increase the user’s willingness to contribute relevant data. This is supported by Flanagin and Metzger’s [26] comments that traditional sources of geographic information such as government institutions, cartographers, or other public institutions are usually considered as credible because they are recognized authorities. Chen et al. [15] also note that a framework provided by the government can contribute to quality assurance, for example, by providing an application or platform to collect the information or even official geodata as a basis. In the event of a disaster, the public can use this framework to quickly collect and analyze data [15]. The basic dataset of house perimeters chosen in this work provides high coverage for places where people have stayed inside a building. However, it does not adequately represent spaces that cannot be mapped in the form of a house perimeter, such as streets, train stations, parks, public squares, and other outdoor locations. The dataset of the district government of Cologne has a high spatial coverage for buildings in the city area but does not guarantee the inclusion of every house perimeter in Cologne. For example, in the city center near Barbarossaplatz, there is a large gap in the dataset (Figs. 10 and 11), as revealed by mapping. Furthermore, certain buildings that could be grouped together as a complex, such as Cologne’s main train station (Fig. 12), are shown in individual polygons. Along with this, it can be helpful to extend the application so that it is possible to select and process several house perimeters simultaneously. Thus, fewer transactions between the client and server are necessary because the update is performed with one request. This also makes the input easier for the user. Simultaneously, no function has yet been implemented to allow post-processing and correction of incorrect records. The house perimeters can be assigned to a building function with the help of the ATKIS object-type catalog. Twenty-six (68.42%) of the 38 house perimeters fall into the residential category. The dominance of residential units can be explained by the fact that since the onset of the pandemic and prescribed measures by the federal government, some activities have been limited to the home. Numerous workers do not necessarily have to go to an office building but can work from home. In addition, nonessential travel can be avoided. Hotels have been closed at times, so they have also been selected infrequently. Less than 50% of all house perimeters are assigned to the residential or residential classes with trade and services in the initial data set, so there is generally a dominance of these categories. However, the classification also reveals weaknesses in the data collection. No attention has been paid to the verticality of buildings. It is not apparent whether a contribution to a residential building with trade and services is related to the residential or service sector. Consequently, the information is lost. Only 12 (31.58%) of the selected house perimeters were purely residential buildings, while 14 (36.84%) also contained trade or service facilities. For the PLZ-8 data, only the corresponding geometries are available. For further research, the associated socioeconomic attributes are particularly important, as well as in connection with building classification. From this aspect, spatial patterns could possibly be derived in the context of VGI-based data collection. To increase transparency, an application for automated data evaluation was developed in this study. This provides data via the REST interfaces, which are immediately visualized. This corresponds to the requirements of Sula [54] that the results should be provided in the form of public channels [54]. For higher transparency, the implementation of a higher number of endpoints is discussed. In particular, geospatial data can be provided increasingly via the API. In addition to the endpoint that provides district-level case counts in the GeoJSON format in this work, interfaces could be implemented in the same way to output district-level or ZIP code-level case counts. With GeoJSON output, authorized administrative bodies could visualize the data independently in a GIS for their own purposes. In general, the output of a wide variety of information via an API is conceivable. Any query that can be made via SQL in Postgres can also be implemented as a REST interface. However, as already mentioned, data protection aspects must be considered. For this, the consent of the users would have to be obtained in advance at the time of collection of data from third parties. This was not the case in this study. Furthermore, notably, no personal data are provided in the API, and it is not possible to draw conclusions about individuals’ data. For example, an interface that outputs all visited house perimeters is unsuitable. It should also be noted that an API and its application must always be comprehensively documented. The developed API offers various interfaces that provide automated results for spatial distribution, among other things. However, in its current alpha version, it is not usable for outsiders. As part of the documentation, all the interfaces must be explained in detail. For example, users have to be able to see which endpoints output which data with which attributes, which requests to the API are generally possible (GET, POST, etc.), or what must be considered for potential transactions, for example, data type and field length in the database. Therefore, the source code must be extended. Especially in implementations of interfaces for update or delete operations, input parameters have to be checked for their validity on the client and server side, such that manipulations by SQL injection are not possible. The modules used in the development provide functions that perform such checks and can be extended if required. In this work, an application for automated data evaluation was developed, especially for transparency purposes. Simultaneously, various authors call for results to be made available via open channels. However, the app for data collection does not refer to open data evaluation. On the one hand, this is due to IT security reasons, and, on the other hand, the automatic evaluation is only to be tested in this work for the time being. It is possible that this approach has contributed to the fact that some visitors to the site were deterred from participating, but they would have been willing to contribute data if they had also been given direct access to results, for example, in the form of the developed API with node.js. In the context of the application for data evaluation, manual corrections may also be necessary, or the SQL query for determining the spatial position of a house perimeter may have to be revised or supplemented. Figure 13 shows a house perimeter that is not clearly located within an area of the Urban Atlas. A pure query using the Postgres function ST_WITHIN is not sufficient here. The house perimeter was not assigned to the polygon of the urban atlas. Alternatively, instead of using further Postgres functions like ST_INTERSECTS, each building can also be represented with the help of a point, for example, the center of the house perimeter polygon, so that a pure mapping via ST_WITHIN would be possible here.

For this, however, further tables must be created in the database or the point representations of a house perimeter must be calculated within the SQL query. In this work, because of the problem of an insufficient SQL query, manual corrections were necessary to determine the correct case numbers. Thus, although the application for automated analysis is functional and provides a visualization of the results, the SQL queries of the endpoints need to be revised so that a correct output of the data is produced. This is possible, for example, using the previously described methodology. Another possible extension that enhances the application is the implementation of notifications when two users have selected the same house ring. In this way, it is directly visible where contact might have existed. At the same time, however, data protection aspects have to be considered, so that it is not possible for others to know who the contact person is. During data collection, users updated several house perimeters twice or thrice within one browser session. Therefore, it may be necessary to improve the user feedback or revise the instructions for recording the data and add the attribute “Number of visits”. This work does not employ an approach that uses Bluetooth technology described in Chapter 2. The full use of Bluetooth functionalities, as in the Corona-Warn-App, has so far been reserved for native apps. However, developers are actually working on implementing a Bluetooth API for browsers. However, these are experimental features [5]. These could be relevant in the future so that users with operating systems other than iOS or Android are not prevented from using them. This would make it possible for at-risk patients with Windows or Huawei operating systems to use the app. The cost factor is of little importance here, all common browsers use JS to manipulate content, so there is no duplication of development work. Furthermore, the extent to which the tracking of the web server (in this work, Nginx) is considered in the data evaluation or to what extent this is restricted or regulated in advance must be taken into account. In the context of data protection, logging of the web server is essential to ensure security for the application and to identify requests to the server with the intention of tapping information or access data and to block these IP addresses. In contrast, the standard logging of the web server also allows conclusions to be drawn about an individual IP address. Access times or frequencies of page calls and requests to the API to generate new tokens or the origin of the application call can be traced. For example, in this work, from the logs, we can trace that users accessed the URL from WhatsApp, Instagram, or Facebook, which are popular social networks in Germany. The applications developed can only be used to a limited extent for contact tracking. Although it is possible to see whether different pseudonyms have updated the same data set and thus whether there was potential contact, it is not possible to clearly determine whether the same person may have updated the same house ring under several pseudonyms and thus falsify the data collection. Furthermore, time was not considered. It is possible that there is a period of several weeks or months between visits to a building by two people, so that the contact cannot be classified as relevant. The time factor, therefore, must be considered in the further development of the application, for example, by specifying the time when the building was visited during processing. Here again, data protection aspects must be considered. If necessary, detailed information about a certain point in time can narrow the group of people, so that it may be possible to draw conclusions about individuals. If the time factor is considered, double or multiple entries of buildings can also be meaningful and plausible, for example, if a building is repeatedly visited on a daily basis. Furthermore, it is difficult to make a statement regarding the validity of the collected data, which is a clear weakness of VGI in the context of health-related, personal data. The dataset can be cleaned for duplicate or multiple selections because of the verification mechanisms used, but it is not possible to verify whether a participant was actually infected or visited a selected building. In other VGI projects, such as the OSM, contributions are always verifiable for third parties. For example, if paths or buildings are digitized, others can see whether they actually exist. However, cases of disease are not physically represented in the landscape; thus, no visual inspection can be performed. This goes hand-in-hand with ethical problems. If measures are derived from such an application, but a large part of the input data is based on false assertions, these are not target-oriented and the credibility of the application (i.e., data integrity) is not given. No valid statement can be made regarding the actual distribution of cases of the disease in urban areas. In general, consideration should be given to supplementing the methods used with additional methods, especially qualitative methods. An example for this could be interviews with test persons who use the tool and voluntarily agree to answer questions about the developed applications. In particular, helpful user experiences can be collected to identify weaknesses.

## Conclusion

### To what extent can a web application for the spatial recording/evaluation of COVID-19 cases in the urban area of Cologne be developed based on open-source products?

A Web GIS can be implemented in a relatively simple manner with the help of open-source software. However, knowledge regarding various markup and scripting languages as well as libraries is necessary. Furthermore, a minimum knowledge of the spatial data standards is required. Software components that have been used in this work, such as Geoserver, OpenLayers, or Postgres, relieve the developer of a lot of development work due to their advanced implementation and ready-made functions or classes. Geoserver also offers a user interface with which geodata can be published in the usual standard formats such as WMS or WFS; thus, less knowledge of programming is required. The high availability of open data on various issues makes it easy to collect information on a voluntary basis. The use of an open-source product ensures easy transferability to other cities, especially in Germany. Spatial data with different resolutions can be stored in the database. The query of the location of the house perimeters is the same; only trigger functions, for example, have to be adapted with regard to their table names. In addition, for cities in developing countries, implementation is possible, provided geodata are available. Nevertheless, one must rely on the motivation of the potential participants. For automated data evaluation, the endpoints of the API must be slightly modified, and SQL queries must be marginally modified. In general, this offers the possibility of making results available to third parties in an uncomplicated manner, considering the relevant data protection regulations. Simultaneously, however, comprehensive documentation must be created for this in the future. The endpoints can then be extended as desired, for example, for the output of results on different spatial scales. For better development, a Docker container should be supplied, including a setup for Postgres and Geoserver with corresponding data.

### What data protection, ethical, and legal aspects must be considered when using VGI and in the context of application development?

Data protection must always be of high priority. Lack of protection can deter people from participating and have devastating consequences in the event of data being accessed, for example, by hackers. Ethical aspects go hand-in-hand with data protection issues, which also have to be considered simultaneously when programming in the context of IT security. In addition, from an ethical perspective, it has to be ensured that no conclusions can be drawn regarding individual persons (groups). Certain standards, such as the use of server certificates (https), must always be ensured when developing corresponding applications. By not using map services from Google, it can be ensured that one is not bound by third-party privacy/usage policies and may not be further used by them. Furthermore, the separation of data collection and data evaluation is important. For future scientific work, a guideline with recommendations for action and concrete application examples for the consideration of data protection and ethical or legal aspects could be developed, which is explicitly valid for VGI-based applications. Especially for the applications developed in this study, the aspect of intervenability is not given enough attention, which is why the future development of corresponding functions must be in the foreground.

## Data Availability

Application: Github-Repository; Data collection: https://github.com/fschmidt56/ma_app; Data evaluation: https://github.com/fschmidt56/ma_dashboard_client; API: https://github.com/fschmidt56/ma_dashboard_server
