# Correction to: Development of a Web GIS for small-scale detection and analysis of COVID-19 (SARS-CoV-2) cases based on volunteered geographic information for the city of Cologne, Germany, in July/August 2020

**DOI:** 10.1186/s12942-021-00295-9

**Published:** 2021-09-29

**Authors:** Fabian Schmidt, Arne Dröge-Rothaar, Andreas Rienow

**Affiliations:** Institute of Geography, Ruhr University Bochum, Universitätsstraße 150, 44780 Bochum, Germany

## Correction to: Int J Health Geogr (2021) 20:40 https://doi.org/10.1186/s12942-021-00290-0

In this article [1], the wrong figures appeared as Listings 1 to 4; the Listings 1, 2, 3 and 4 should have been appeared as shown.

The in-text reference citations has been arranged in numerical ascending order and the corresponding list has been arranged accordingly.

**Listing 1:**
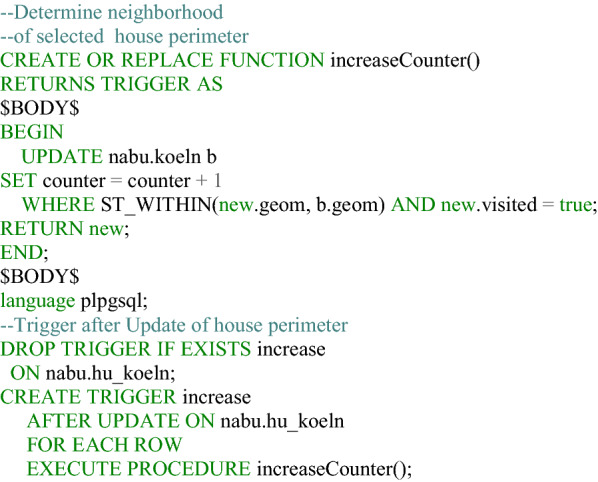
Function to determine the location of a house perimeter in an urban area

**Listing 2:**
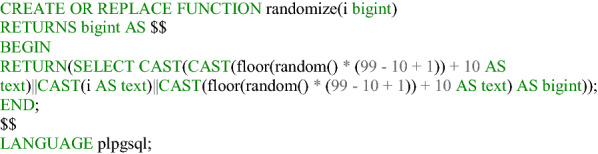
Function to generate random ID in Postgres

**Listing 3:**
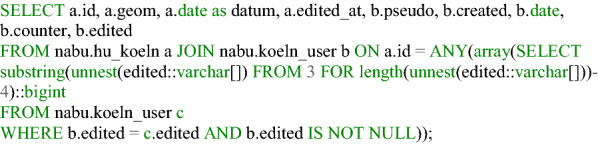
Decryption of the random ID and assignment of the original ID to identify the edited house perimeters of a user

**Listing 4:**
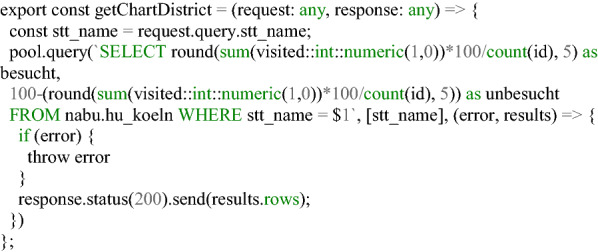
Code snippet for the SQL query in Node via a REST interface
